# Mathematical skills in deaf and hard-of-hearing children: A systematic review

**DOI:** 10.1371/journal.pone.0358324

**Published:** 2026-09-16

**Authors:** Carla Laria, Nicola Serra, Annarosa Trofa, Angelica Rodio, Giuseppe Chiarella, Pasquale Viola, Federica D’Ambrosio Geremicca, Emma Landolfi, Rita Malesci, Anna Rita Fetoni

**Affiliations:** 1 Audiology Section, Department of Neuroscience and Reproductive Sciences and Dentistry, University of Naples Federico II, Naples, Italy; 2 Unit of Audiology, Phoniatrics and Vestibology, Regional Centre for Cochlear Implants and ENT Diseases, Department of Experimental and Clinical Medicine, University Magna Graecia, Catanzaro, Italy; 3 Hearing and Balance Unit, Department of Head and Neck, Federico II University Hospital, Naples, Italy; The University of Jordan, JORDAN

## Abstract

**Introduction:**

Mathematical competencies are essential for both academic achievement and everyday functioning. Research investigating mathematical skills in deaf and hard of hearing (DHH) individuals has produced heterogeneous findings, indicating domain-specific performance patterns and the influence of linguistic and cognitive factors. Recent evidence further suggests that these differences may emerge early in development. This systematic review aims to evaluate mathematical competencies in DHH individuals and to contextualize their mathematical profile in relation to developmental dyscalculia.

**Methods:**

A systematic review of observational studies was conducted in accordance with PRISMA 2020 guidelines. Studies were selected based on the PICOS framework, including participants under 18 years of age with congenital or acquired hearing loss. Comparisons involved hearing peers with or without specific learning disorders, and outcomes were based on direct assessments of mathematical abilities or related cognitive processes. A comprehensive literature search was performed in PubMed, Scopus, and Web of Science between March 31 and April 3, 2026, including all studies published up to the search date. In addition, relevant studies were identified through manual screening of reference lists. Methodological quality was assessed using the Joanna Briggs Institute (JBI) critical appraisal tools. Due to the heterogeneity of the included studies, results were synthesised narratively rather than through meta-analysis.

**Results:**

The twelve included studies showed substantial variability in mathematical performance among DHH individuals. Lower achievement was more consistently observed in tasks with high linguistic demands, such as word problem solving, measurement, and mathematical reasoning, whereas procedural and visually supported tasks were relatively preserved. Evidence from both preschool and school-aged populations suggests that these differences may emerge early in development and remain observable across different developmental stages. Linguistic proficiency and access to a fully accessible language emerged as key factors associated with mathematical outcomes. Additional sources of variability included educational setting, communication modality, age of language acquisition, and hearing-related characteristics. Overall methodological quality was generally characterized by moderate methodological concerns, most commonly related to limited control of confounding variables, incomplete reporting of measurement validity, and small sample sizes.

**Conclusion:**

Mathematical difficulties in deaf and hard of hearing (DHH) individuals appear to be domain-specific rather than global and appear to be associated with linguistic access and educational experiences. Evidence from both preschool and school-aged populations suggests that these differences may emerge early in development and remain evident across studies involving different age groups. These findings highlight the importance of considering language-related factors in both the assessment and instruction of mathematics in DHH learners.

## Introduction

Mathematical learning difficulties encompass a heterogeneous spectrum of impairments in the acquisition of numerical and arithmetic competencies that cannot be accounted for by intellectual disability, neurological pathology, or inadequate educational exposure. Among these conditions, developmental dyscalculia (DD) is one of the most extensively characterized. DD is defined by persistent deficits in core numerical processing and arithmetic reasoning despite intact intellectual functioning and adequate schooling [1,2]. Given the centrality of numerical competence in modern societies, DD is associated with significant consequences for everyday functioning, academic achievement, and long-term occupational outcomes [3]. However, the absence of a universally accepted, psychometrically grounded definition has led to substantial heterogeneity in diagnostic criteria and cognitive phenotypes, contributing to inconsistent findings across the literature [4].

DD is considered here as a conceptual comparator to help distinguish domain-specific numerical deficits from difficulties that may be more strongly influenced by linguistic and educational factors in DHH populations.

Converging evidence further indicates that deaf and hard of hearing (DHH) children frequently

show lower academic attainment than their hearing peers, particularly in literacy and mathematics [5,6]. Linguistic deprivation—arising from delayed, restricted, or qualitatively limited access to spoken and/or signed language—has been consistently identified as a primary mechanism underlying these disparities [7]. Language is critically implicated in the development of numerical cognition, especially in domains requiring symbolic representation, arithmetic fact retrieval, and problem solving [8]. Accordingly, reduced access to a fully accessible language during early development may constrain the acquisition of foundational mathematical concepts in DHH populations.

A growing body of research has also examined domain-general cognitive mechanisms that may contribute to mathematical performance in DHH children. In particular, working memory deficits have been documented in many children with hearing loss, potentially limiting their capacity to maintain and manipulate numerical information and to execute multi-step computations [9,10]. Comparative investigations between DHH and hearing populations have yielded mixed results: some studies report global impairments in mathematical achievement among DHH learners [11], whereas others suggest that performance is more selectively affected and moderated by factors such as language proficiency, communication modality, and educational context [12]. This variability underscores the need for a systematic and integrative synthesis of the existing evidence.

Understanding the relationship between hearing status and mathematical development is of critical importance from educational and clinical perspectives.

Despite the growing body of research in this area, a clear and systematic synthesis of evidence specifically addressing domain-specific mathematical skills in DHH children and adolescents, and their relationship with linguistic and cognitive factors, is currently lacking.

The present systematic review aims to comprehensively evaluate mathematical competencies in deaf and hard of hearing (DHH) children and to compare their performance with that of hearing peers, including those with specific learning disorders such as developmental dyscalculia (DD). By integrating findings across heterogeneous populations and observational methodological approaches, this review seeks to characterize patterns of mathematical performance observed in DHH children and adolescents and to examine the linguistic and domain-general cognitive factors associated with these differences. Ultimately, this work aims to provide an evidence base to inform educational practice, refine clinical assessment, and guide the development of targeted, evidence-based interventions for DHH children.

## Materials and methods

### Study design

This systematic review was conducted in accordance with the Preferred Reporting Items for Systematic Reviews and Meta-Analyses (PRISMA) 2020 guidelines [13]. The review protocol defined the research question, eligibility criteria, search strategy, study selection procedures, data extraction, and risk-of-bias assessment prior to the literature search. The study protocol was registered in the OSF Registries on April 27, 2026 (https://osf.io/eqxmz/overview; DOI: https://doi.org/10.17605/OSF.IO/EQXMZ). The PRISMA 2020 checklist is provided in the Supplementary Materials (S1 Checklist).

Although registration occurred after completion of the initial literature search, the review was conducted according to a predefined protocol and no major deviations were introduced during study selection, data extraction, or methodological appraisal. Nevertheless, retrospective registration represents a methodological limitation, as it provides less transparency than prospective registration and may reduce the ability to fully verify that review methods were established before the review process commenced.

The research question for this systematic review was: “What is the association between deafness and the development of mathematical skills in deaf and hard-of-hearing (DHH) children and adolescents?”.

### Inclusion criteria and eligibility

The study selection process followed the PICOS framework (Population, Exposure, Comparator, Outcome, Study design), in accordance with PRISMA 2020 recommendations [13]. Eligible studies met the following criteria:

1. *Population:* Deaf or hard-of-hearing (DHH) children under the age of 18.
2. *Exposure:* Presence of congenital or acquired hearing loss.
3. *Comparator:* Hearing peers, with or without specific learning disorders, when available; studies without a comparison group were also eligible if they included direct assessment of mathematical skills in DHH participants.
4. *Outcome:* Direct assessment of mathematical skills, including numeracy, arithmetic performance, and domain-specific mathematical abilities, as well as related cognitive processes (e.g., working memory, numerical representation, and problem-solving abilities) when directly linked to mathematical performance.
5. *Study design:* Observational studies, including cross-sectional, cohort, and case-control designs. Both quantitative and qualitative observational studies were considered eligible, provided they examined mathematical skills or related processes in DHH children.

The following study designs were excluded: randomized controlled trials, clinical trials, intervention studies, study protocols, meta-analyses and narrative reviews, case reports, conference papers, letters, commentaries, theses, and posters. These study designs were excluded because the aim of this review was to examine the association between hearing status and mathematical performance in naturalistic conditions. Such designs involve structured interventions that may influence participants’ performance and therefore limit comparability when assessing baseline mathematical competencies.

These inclusion criteria were intentionally defined to ensure methodological consistency across studies, prioritizing observational designs and direct assessments of mathematical skills. Although this approach may have limited the number of eligible studies, it was adopted to enhance the internal validity and interpretability of the findings.

### Search strategy

To identify studies examining mathematical skills in deaf and hard-of-hearing (DHH) children, a comprehensive literature search was conducted in the following electronic databases: PubMed (National Center for Biotechnology Information, Bethesda, MD, USA), Scopus, and Web of Science.

The search was performed between March 31 and April 3, 2026, and included all studies published up to the search date. Search strategies combined controlled vocabulary (e.g., MeSH terms) and free-text keywords related to deafness, hearing loss, mathematical skills, numeracy, and arithmetic performance, following PRISMA 2020 recommendations [13]. The search strategy was adapted for each database according to its specific indexing system and search interface. Only studies published in English were considered. Filters related to observational study designs and publication types were applied where supported by the database interfaces, in accordance with the predefined eligibility criteria. In particular, non-primary research articles, including reviews, conference papers, study protocols, book chapters, and other non-eligible publication types, were excluded during the initial screening phase. In addition, the reference lists of included studies and relevant articles were systematically screened to identify further eligible records, as a complementary strategy to minimize the risk of missing relevant studies.

The complete search strings used for each database are reported in Table 1 and represent the full search strategies applied.

**Table 1:** Database, search strings and number of articles found.

Database	Search Strings	Number of results
PubMed	(“Deaf”[Mesh] OR “Hearing Loss”[Mesh] OR deaf* OR “hard of hearing” OR “hearing impaired” OR “sign language users”) AND (“Mathematics”[Mesh] OR mathematics OR numeracy OR arithmetic OR “math skills” OR “number processing” OR calculation OR “mathematical ability”) AND (child* OR adolescen* OR student*) AND (“Observational Study”[Publication Type] OR “Comparative Study”[Publication Type] OR “Evaluation Study”[Publication Type] OR “Case-Control Studies” OR “Cohort Studies” OR “Cross-Sectional Studies”)	352
Scopus	(deaf* OR “hard of hearing” OR “hearing impaired” OR “hearing loss” OR “sign language users”) AND (mathematics OR numeracy OR arithmetic OR “math skills” OR “number processing” OR calculation OR “mathematical ability”) AND (child* OR adolescen* OR student*) AND (“observational study” OR “comparative study” OR “evaluation study” OR “case-control” OR “cohort” OR “cross-sectional”)	98
Web of Science	(deaf* OR “hard of hearing” OR “hearing impaired” OR “hearing loss” OR “sign language users”) AND (mathematics OR numeracy OR arithmetic OR “math skills” OR “number processing” OR calculation OR “mathematical ability”) AND (child* OR adolescen* OR student*) AND (“observational study” OR “comparative study” OR “evaluation study” OR “case-control” OR “cohort” OR “cross-sectional”)	129

### Study selection and data extraction

Two authors with at least five years of experience in systematic review methodology (A.T., C.L.) independently screened the titles and abstracts retrieved through the search strategy, focusing on studies examining the relationship between hearing loss and mathematical skills in students. During the first phase, both reviewers independently assessed titles and abstracts and selected potentially relevant records using an intentionally inclusive approach and subsequently evaluated the full texts of all eligible articles to determine their suitability for inclusion. Any disagreements were first discussed and resolved through consensus between the two reviewers. When consensus could not be reached, a third reviewer with expertise in biostatistics (N.S.) was consulted. For the study authored by members of the review team (Laria et al., 2025), screening, data extraction, and methodological quality assessment were conducted by reviewers who were not involved in the original study to ensure objectivity.

Data extraction was performed independently by the same two reviewers using a predefined data extraction form. Extracted information included study characteristics, sample size, study design, assessment tools, and main outcomes. Any disagreements were resolved through discussion and, when necessary, by consultation with the third reviewer.

The extracted dataset underlying the review findings is available in the Supplementary Materials (file S1 Dataset).

### Methodological quality assessment

Two reviewers with expertise in systematic review methodology and critical appraisal independently assessed the included studies using the JBI Critical Appraisal Checklists. A third reviewer, a biostatistician (N.S.), was consulted to resolve any discrepancies. The JBI tool provides design-specific checklists, which were applied according to study design: the Checklist for Analytical Cross-Sectional Studies, the Checklist for Case-Control Studies, and the Checklist for Qualitative Research [14]. Each checklist includes items evaluating key methodological domains such as clarity of inclusion criteria, validity and reliability of measurement tools, identification and management of confounding factors, appropriateness of statistical analyses, and completeness of outcome reporting.

A formal assessment of the overall certainty of evidence using the GRADE approach was not performed. This decision was made because the included studies were highly heterogeneous with respect to study design, participant characteristics, assessment instruments, mathematical domains evaluated, and outcome reporting. In addition, the limited number of studies available within individual mathematical domains and the absence of comparable quantitative outcome measures precluded a meaningful assessment of the certainty of evidence across outcomes. Therefore, the overall strength of the evidence was considered through narrative synthesis, taking into account methodological quality, consistency of findings, and limitations identified across the included studies.

### JBI critical appraisal tools

The JBI Critical Appraisal Checklist for Analytical Cross Sectional Studies was applied to ten quantitative observational studies: Titus (1995) [15]; Zarfaty et al. (2004) [16]; Rodríguez Santos et al. (2014) [17]; Blatto Vallee et al. (2007) [18]; Lang and Pagliaro (2007) [19]; Ross and Hoemann (1975) [20]; Palma et al. (2010) [21]; Kritzer KL. (2009) [22]; Pagliaro CM (2013) [23]; and Swanwick R (2005) [24].

Notably, two distinct studies by Kritzer (2009) were included: one quantitative [22] and one qualitative [25], which were assessed using different JBI tools.

This checklist includes eight items assessing: (1) clarity of inclusion criteria for the sample, (2) detailed description of study participants and setting, (3) validity and reliability of exposure measurement, (4) use of objective and standardized criteria for outcome measurement, (5) identification of potential confounding factors, (6) description of strategies used to address confounding factors, (7) validity and reliability of outcome measurement, and (8) appropriateness of the statistical analyses employed.

The JBI Critical Appraisal Checklist for Case-Control Studies was applied to the study by Laria et al. (2025) [26].

This checklist comprises ten items assessing: (1) comparability of cases and controls, (2) appropriateness of matching or statistical adjustment, (3) use of the same criteria for identification of cases and controls, (4) validity and reliability of exposure measurement, (5) consistent measurement of exposure for cases and controls, (6) identification of confounding factors, (7) strategies to address confounding factors, (8) validity and reliability of outcome assessment, (9) appropriate statistical analysis, and (10) adequacy of follow-up or exposure period where applicable.

The JBI Critical Appraisal Checklist for Qualitative Research was used to evaluate the qualitative observational study by Kritzer (2009) [25].

This checklist comprises ten items examining: (1) congruity between the stated philosophical perspective and the research methodology, (2) congruity between the methodology and the research question or objectives, (3) congruity between the methodology and data collection methods, (4) congruity between the methodology and data analysis, (5) congruity between the methodology and interpretation of results, (6) positioning of the researcher culturally or theoretically, (7) consideration of the influence of the researcher on the research process, (8) adequate representation of participants’ voices, (9) adherence to ethical standards and evidence of ethical approval, and (10) coherence between the data analysis and the conclusions drawn.

Each item was rated as Yes, No, Unclear, or Not applicable. Methodological quality was interpreted through a qualitative assessment of individual JBI appraisal domains, including participant selection, measurement validity, management of confounding factors, and appropriateness of statistical analyses. Item-level ratings are reported in Table 3 to ensure transparency of the appraisal process.

Overall methodological appraisal categories (e.g., low or moderate methodological concerns) were derived through qualitative consideration of the methodological strengths and limitations identified across JBI domains, rather than through numerical scoring or predefined thresholds. Studies were classified as presenting low methodological concerns when no substantial methodological limitations were identified across key methodological domains and when any remaining limitations were unlikely to materially affect interpretation of the findings. Studies presenting one or more relevant methodological limitations in these domains were classified as having moderate methodological concerns. Particular attention was given to limitations related to confounding control, validity and reliability of outcome measures, and completeness of methodological reporting, as these factors were considered most likely to influence the interpretation of study findings and are directly related to the internal validity and interpretability of observational research. This qualitative categorization was introduced as an interpretative framework to facilitate a consistent and transparent synthesis of methodological quality across studies and does not reflect an official scoring system defined by the Joanna Briggs Institute.

Given the inclusion of both qualitative and quantitative studies, different JBI appraisal tools were applied according to study design. Although these tools assess distinct methodological aspects, methodological judgments were derived through qualitative appraisal of key methodological domains to facilitate a consistent interpretation across studies.

## Results

Overall, the database search yielded 579 records (352 from PubMed, 98 from Scopus, and 129 from Web of Science). In addition, three records were identified through manual screening of reference lists, resulting in a total of 582 records. After removing duplicate records (n = 2) and applying initial exclusion criteria (n = 40), a total of 540 records were screened by title and abstract. A total of 500 records were excluded because they did not meet the inclusion criteria (e.g., non-mathematical outcomes, adult populations, or non-observational study designs).

A total of 40 full-text articles were assessed for eligibility. Among these, 28 were excluded for the following reasons: inclusion of adult populations or absence of participants under 18 years of age (n = 11), lack of direct assessment of mathematical outcomes (n = 9), mathematical performance as a secondary outcome (n = 4), intervention or training designs (n = 3), and mixed samples without separate analysis for DHH participants (n = 1). Finally, 12 studies met all eligibility criteria and were included in the systematic review.

**Fig 1:**
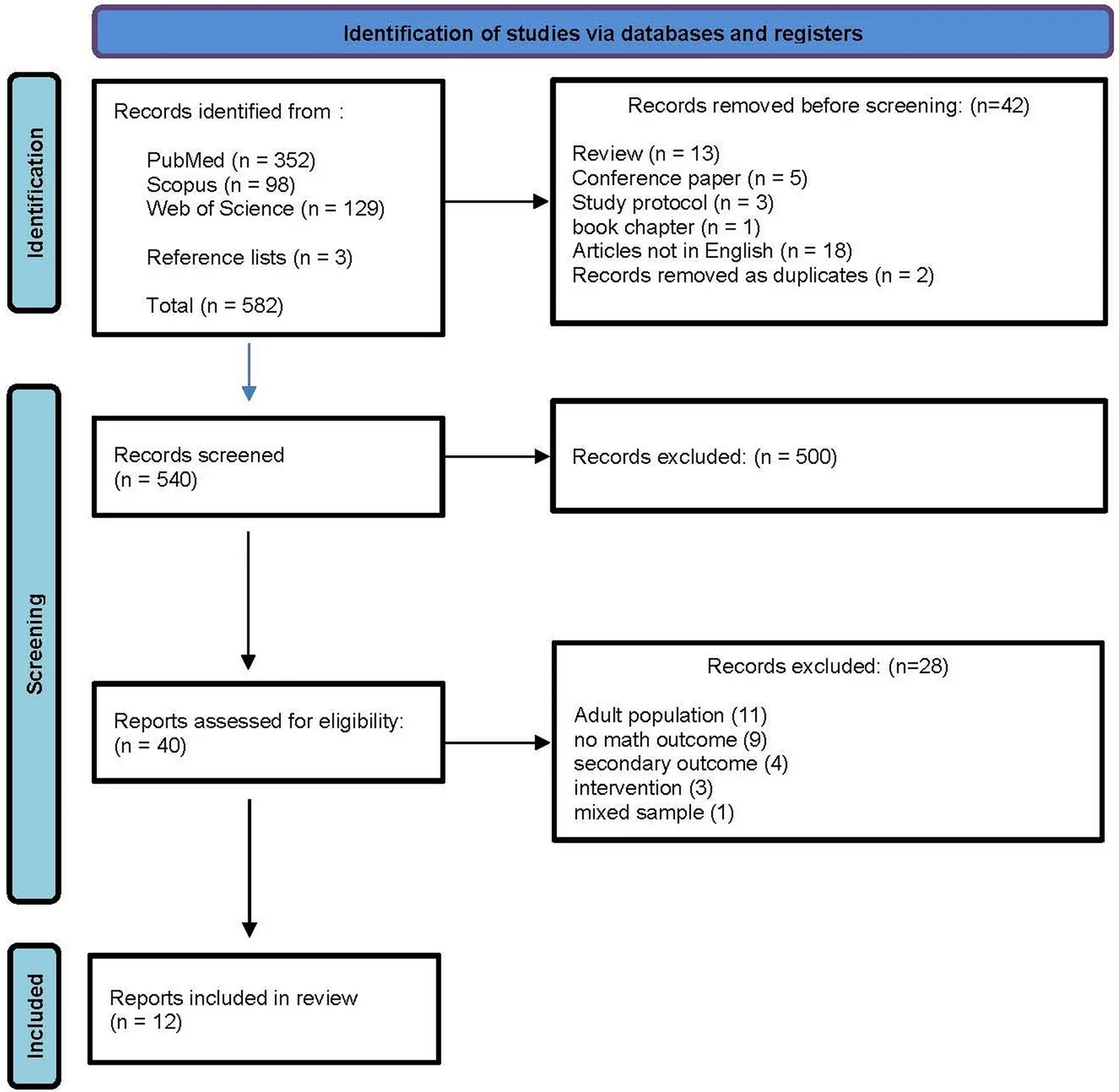
PRISMA flow diagram 2020. The flowchart displays article search and selection.

The details of the research performed are shown in the flowchart in Fig 1.

In Table 2 we reported the description of all included papers, describing, first author, title, journal, publication year, database, aim of the study, type of study, sample size, tools used and study results.

**Table 2:** Description of included articles.

Ref	1) First author, 2) Country 3) Journal, year	Aim of the study	1) Study design, 2) Sample size, 3) Population	Results
15	Titus JC. USA Am Ann Deaf, 1995	To examine the understanding of fractional numbers among deaf and hard of hearing students and identify potential difficulties compared to hearing peers.	**Study design:**Cross-sectional comparative study **Sample size:**47 students (21 deaf and hard-of-hearing, 26 hearing) **Population:**Deaf and hard-of-hearing and hearing students aged 10–16 years, enrolled in grades 3–10, with prior experience in fractions; DHH participants with early-onset hearing loss from mixed educational placements, compared with hearing peers from regular classrooms	Deaf and hard-of-hearing students showed lower performance in fraction understanding compared to hearing peers, particularly in conceptual aspects, suggesting delays in higher-level mathematical reasoning.
16	Zarfaty Y, et al., UK Deaf Stud Deaf Educ. 2004	To investigate whether and how deafness affects young children’s numerical abilities by comparing their performance with hearing peers on spatial and temporal number tasks.	**Study design:**Cross-sectional comparative study **Sample size:**20 children (10 deaf and 10 hearing) **Population:**Children aged 31–54 months (mean ≈ 39.8 months); 10 deaf children (mostly profoundly deaf, majority with cochlear implants) and 10 age-matched hearing children	Deaf children performed similarly to hearing peers in spatial numerical tasks but showed lower performance in temporal tasks, suggesting a specific role of auditory experience in temporal number processing.
17	José Miguel Rodríguez‑Santos, et al, Spain Am Ann Deaf. 2014 Spring	To examine whether deaf and hard of hearing children differ from hearing peers in processing quantity information, using symbolic (Arabic numerals) and nonsymbolic (dot and hand arrays) magnitude comparison tasks	**Study design:**Cross‑sectional comparative study. **Sample size:**20 children total (10 deaf/hard of hearing children and 10 normally hearing children). **Population:**Prelocutive deaf and hard of hearing children and age‑matched normally hearing children.	Deaf and hearing children showed comparable magnitude representation in symbolic and nonsymbolic tasks, but DHH children exhibited slower response times in symbolic processing, indicating delayed access to numerical representations.
18	Blatto-Vallee G, et al., USA J Deaf Stud Deaf Educ. 2007 Fall	To examine how deaf and hearing students use visual–spatial representations when solving mathematical problems, and how these representations relate to problem-solving performance.	**Study design:** Cross-sectional comparative study across educational levels (middle school, high school, college). **Sample size:** 305 participants: 149 deaf students, 156 hearing students. **Population:** Deaf and hearing students at middle school, high school, and college age; both genders included	Hearing students outperformed deaf students in mathematical problem solving, with performance influenced by the type of visual–spatial representation used.
19	Lang H et al., USA J Deaf Stud Deaf Educ. 2007	To identify which characteristics of mathematics terms (imagery, familiarity, concreteness, signability) best predict recall performance among deaf high school students, and to explore implications for teaching.	**Study design**: Cross‑sectional correlational study examining predictors of recall within a sample of deaf students. **Sample size:** 18 deaf/hard-of-hearing high-school students; teacher ratings and recall data were also collected from 75 teachers **Population**: Deaf and hard-of-hearing high-school students enrolled in two residential educational programs in the United States, currently attending or having previously completed geometry courses.	Imagery and familiarity were the strongest predictors of geometry-term recall among DHH students; recall was higher for highly imageable, familiar, and easily signable terms.
20	B. M. Ross, H. Hoemann USA Genet Psychol Monogr. 1975	Compare probability concept attainment (formal operations) in deaf vs hearing adolescents	**Study Design:** Comparative observational study **Sample size:** 466 adolescents (180 deaf, 286 hearing) **Population:** Deaf and hearing adolescents, with additional preadolescent groups included in some experiments	Deaf adolescents performed similarly to hearing peers on simple probability tasks, but showed more errors, less consistent rule use, and a 2–3‑year lag on complex probability concepts.
21	Silvia Palma et al., Italy Cochlear Implants International (2010 Jun)	Investigate numerical intelligence, verbal competence, and overall cognitive profile in preschool children with cochlear implants	**Study Design:** Cross-sectional comparative study **Sample size:** 57 children (17 preschool children with unilateral cochlear implants and 40 age- and sex-matched hearing controls). **Population:** Preschool children aged 31–72 months with profound hearing loss (>90 dB HL) who underwent unilateral cochlear implantation, compared with age- and sex-matched normally hearing preschool children	Lower numerical intelligence in number comparison tasks; fluid reasoning correlated with overall IQ, highlighting integration of visual-spatial and cognitive skills
22	Kritzer KL. USA J Deaf Stud Deaf Educ, 2009	To examine young deaf children’s early informal and formal mathematical knowledge before the onset of formal schooling, as measured by the Test of Early Mathematics Ability‑3 (TEMA‑3), and to identify the mathematical areas that are most challenging for them.	**Study design:** Descriptive observational study. **Sample size:**29 deaf children participated in the study; TEMA‑3 was administered to all 29 children, and one child was later excluded from the analysis as an outlier. **Population:**Deaf children aged 4–6 years from seven schools for the deaf in the United States, with no additional disabilities, and from homes in which American Sign Language or spoken English was the primary language	Deaf preschool children showed lower mathematical performance than normative expectations, particularly in language-mediated tasks, indicating early emerging delays.
23	Pagliaro CM. USA J Deaf Stud Deaf Educ, 2013	To describe the mathematical performance of preschool-aged deaf and hard-of-hearing children and identify areas of strength and weakness across different mathematical domains.	**Study design:** Observational cross-sectional study (pre-intervention baseline assessment) **Sample size:** 20 children **Population:** Deaf and hard-of-hearing children aged 3–5 years enrolled in early intervention programs in the United States	Preschool DHH children demonstrated overall lower mathematical performance, with greater difficulties in language-related and problem-solving tasks, while some domains appeared relatively preserved.
24	Swanwick R. UK Deafness & Education International, 2005	To explore the factors underlying mathematical underachievement in deaf students by analysing their performance on national mathematics tests and identifying barriers related to language, cognition, and educational experience.	**Study design:** Comparative observational study. **Sample size:** 126 test papers from deaf students **Population:** Deaf students aged around 14 years (Key Stage 3) in the UK, with comparison to hearing peers’ national test data	Deaf students showed lower performance in linguistically demanding mathematical tasks, while routine computational skills were relatively preserved.
25	Kritzer KL USA Am Ann Deaf. 2009	To explore how families with young deaf children mediate and support the development of mathematically based concepts within naturalistic everyday environments.	**Study design:**Qualitative observational study **Sample size:**6 deaf children (selected from a larger sample of 29) **Population:**Deaf preschool children (aged ~4–6 years) with no additional disabilities, recruited from schools for the deaf in the United States and observed in naturalistic home environments interacting with their families	Parents varied in how they incorporated mathematically based concepts (e.g., number, quantity, time, categorization) into everyday activities; children whose parents used more purposeful mediation showed richer engagement with mathematical concepts
26	Carla Laria et al., Italy Hearing Balance and Communication. 2025	This study aims to assess the mathematical skills in children with and without HL through the standardized BDE-2 test and to explore the potential correlations between audiological parameters and cognitive-mathematical performance.	**Study design:** A case–control study **Sample size:** 37 children aged between 9 and 15 years: 25 with bilateral sensorineural hearing loss (study group) and 12 normal-hearing peers (control group). **Population:** Children aged 9–15 years with bilateral sensorineural hearing loss and age-matched hearing controls	Children with hearing loss showed significantly lower performance in calculation and overall mathematical scores, while basic number skills were relatively preserved and unrelated to hearing loss severity.

### Methodological quality assessment

The methodological quality of the twelve included studies was assessed using the Joanna Briggs Institute (JBI) Critical Appraisal Tools, applying the checklist most appropriate to each study design. Specifically, the JBI Analytical Cross-Sectional Checklist (8 items) was used for cross-sectional and comparative observational studies, the JBI Case-Control Checklist (10 items) for the case-control study, and the JBI Checklist for Qualitative Research (10 items) for the qualitative study.

For studies assessed with the JBI Analytical Cross-Sectional Checklist, the reported items were: I1, inclusion criteria clearly defined; I2, study subjects and setting described in detail; I3, exposure measured in a valid and reliable way; I4, objective and standard criteria used for measurement of the condition; I5, confounding factors identified; I6, strategies to deal with confounding factors stated; I7, outcomes measured in a valid and reliable way; and I8, appropriate statistical analysis used.

For the JBI Case-Control Checklist, the reported items were: I1, groups comparable other than the presence of disease/exposure; I2, cases and controls appropriately matched; I3, same criteria used for identification of cases and controls; I4, exposure measured in a standard, valid, and reliable way; I5, exposure measured in the same way for cases and controls; I6, confounding factors identified; I7, strategies to address confounding factors stated; I8, outcomes assessed in a standard, valid, and reliable way; I9, exposure period sufficiently long to be meaningful; and I10, appropriate statistical analysis used.

For the JBI Checklist for Qualitative Research, the reported items were: I1, congruity between the stated philosophical perspective and the research methodology; I2, congruity between the research methodology and the research question or objectives; I3, congruity between the research methodology and the methods used to collect data; I4, congruity between the research methodology and the representation and analysis of data; I5, congruity between the research methodology and the interpretation of results; I6, statement locating the researcher culturally or theoretically; I7, consideration of the influence of the researcher on the research and vice versa; I8, adequate representation of participants and their voices; I9, ethical approval or evidence of ethical considerations; and I10, conclusions supported by the analysis and interpretation of the data.

The appraisal results are presented in Table 3, which reports the individual checklist items and their ratings (Yes, No, or Unclear) for each study, together with methodological notes that support interpretation of the appraisal findings.

**Table 3:** Methodological appraisal of included studies using the JBI Critical Appraisal Tools. Individual checklist items are reported as Yes (Y), No (N), or Unclear (U).

First author (year)	JBI tool used	Items:	Overall methodological appraisal
Titus JC. (1995) [15]	JBI Analytical Cross‑Sectional	**I1:** Inclusion criteria were clearly defined (Y)	Moderate methodological concerns
		**I2:** Participants, age, study setting, and sample characteristics were described in detail. (Y)	
		**I3:** Hearing status was defined using explicit criteria. (Y)	
		**I4:** The assessment instrument was derived from the Rational Number Project; however, formal validity and reliability data were not reported in the study. (U)	
		**I5:** Potential confounding factors were identified and discussed. (Y)	
		**I6:** No specific strategies were reported to control for confounding factors. (N)	
		**I7**: Outcomes were measured consistently across all participant groups. (Y)	
		**I8:** Appropriate statistical analyses were used, including ANOVA and post-hoc comparisons. (Y)	
Zarfaty et al. (2004) [16]	JBI Analytical Cross‑Sectional	**I1:** Inclusion criteria were clearly defined. The study included deaf and hearing children aged 31–54 months, with explicit group allocation based on hearing status. (Y)	Moderate methodological concerns
		**I2:** Participants, age, study setting, and sample characteristics were described in detail. The authors reported age, sex distribution, degree of hearing loss, educational setting, and hearing status of participants. **(Y)**	
		**I3:** Hearing status was measured using objective and explicit criteria. Participants were classified as deaf or hearing, and the severity of hearing loss (profound or moderate) was reported. **(Y)**	
		**I4:** Standard criteria were used for outcome measurement; however, no formal validity or reliability data for the experimental number-representation task were reported. The task was adapted from established paradigms (Piaget; Saxe et al.), but psychometric properties were not provided. **(U)**	
		**I5:** Potential confounding factors were identified and discussed. The authors considered factors such as spatial versus temporal presentation, educational experiences, counting skills, and memory demands. **(Y)**	
		**I6:** No specific statistical or design strategies were reported to control for confounding variables beyond matching groups on age and sex. **(N)**	
		**I7**: Outcomes were measured consistently across all participants. All children completed the same 24 trials under identical experimental conditions, and responses were recorded using the same procedures. **(Y)**	
		**I8:** Appropriate statistical analyses were used, including analyses of variance (ANOVA) and Tukey HSD post-hoc tests to compare groups and conditions. **(Y)**	
Rodríguez‑Santos et al. (2014) [17]	JBI Analytical Cross‑Sectional	**I1:** Inclusion criteria were clearly defined. The study specified eligibility criteria for both groups, including hearing status, age range, language background, absence of additional disabilities, and school placement. **(Y)**	Low methodological concerns
		**I2:** Participants, setting, and sample characteristics were described in detail. The authors reported age, sex, degree of hearing loss, etiology, hearing devices, educational setting, language background, and matching procedures for the control group**. (Y)**	
		**I3:** Hearing status was measured using objective and explicit criteria. Degree of hearing loss, age at diagnosis, type of amplification device, and cochlear implantation history were reported for all participants in the experimental group. **(Y)**	
		**I4:** Outcome measures were described and applied consistently; however, formal psychometric evidence regarding validity and reliability of the symbolic and nonsymbolic comparison tasks was not reported in the article. The tasks were adapted from previous research. **(U)**	
		**I5:** Potential confounding factors were identified and considered. The authors controlled for age, nonverbal intelligence, visual memory, language comprehension, and developmental conditions through participant matching and eligibility criteria. **(Y)**	
		**I6:** Strategies to deal with confounding factors were reported. A matched control design was used, and groups were statistically comparable on major cognitive variables before analysis. **(Y)**	
		**I7**: Outcomes were measured in a valid and reliable manner for all participants. Standardized computerized procedures were used for stimulus presentation and response-time recording, and identical protocols were applied across groups. **(Y)**	
		**I8:** Appropriate statistical analyses were used, including repeated-measures ANOVA, effect size estimation (partial η²), post-hoc corrections, normality checks, and adjustments for violations of sphericity. **(Y)**	
Blatto‑Vallee et al. (2007) [18]	JBI Analytical Cross‑Sectional	**I1:** Inclusion criteria were clearly defined. The study specified participant groups according to hearing status and educational level (middle school, high school, associate degree, and baccalaureate programs), with clear descriptions of group membership and recruitment settings. **(Y)**	Moderate methodological concerns
		**I2:** Participants, study setting, and sample characteristics were described in detail. The authors reported sample size, age, gender distribution, educational level, and degree of hearing loss (PTA) for deaf participants. **(Y)**	
		**I3:** Hearing status was measured using objective and explicit criteria. Degree of hearing loss was documented using pure-tone average (PTA) values, allowing clear classification of participants as deaf or hearing. **(Y)**	
		**I4:** Outcomes were measured using established instruments, including the adapted Mathematical Processing Instrument (MPI), the Primary Mental Abilities Spatial Relations Test, and the Revised Minnesota Paper Form Board Test. Inter-rater reliability for coding visual–spatial representations was reported and was high (.96–.97). However, psychometric information for the adapted MPI version used in this study was not fully reported. **(Y)**	
		**I5:** Potential confounding factors were only partially identified and discussed. The authors considered educational level, hearing status, visual–spatial abilities, and language-related factors but acknowledged that nonverbal intelligence and sign-language proficiency were not controlled**. (Y)**	
		**I6:** No specific strategies were implemented to control for confounding factors. Analyses examined relationships between variables, but participant matching or statistical adjustment for major confounders such as cognitive ability or language proficiency was not reported. **(N)**	
		**I7**: Outcomes were measured in a valid and reliable way for all participants. Standardized administration procedures were used, scoring criteria were clearly defined, and inter-rater reliability for representation coding was excellent. **(Y)**	
		**I8:** Appropriate statistical analyses were used, including ANOVA, post-hoc comparisons, correlation analyses, multiple regression models, and R² increment testing. **(Y)**	
Lang H et al., (2007) [19]	JBI Analytical Cross‑Sectional	**I1:** Inclusion criteria were clearly defined. The study specified that participating deaf students were high school students enrolled in residential programs who were currently studying or had previously studied geometry. Teacher participants were also categorized according to mathematics background and sign-language experience. **(Y)**	Low methodological concerns
		**I2:** Participants, study setting, and sample characteristics were described in detail. The authors reported sample sizes, educational settings, age of the deaf students, and criteria used to classify teachers according to mathematics qualifications and sign-language experience. **(Y)**	
		**I3:** Hearing status was defined using explicit criteria. Participants were identified as deaf/hard-of-hearing students or deaf/hearing teachers, although audiological measures or degree of hearing loss were not reported. **(Y)**	
		**I4:** Outcome measures were clearly described, and reliability estimates were reported for all rating scales. However, formal validity evidence for the study-specific measures was not fully documented.**(U)**	
		**I5:** Potential confounding factors were identified and considered. The study examined mathematics background, hearing status, and sign-language experience as variables that could influence recall and ratings. **(Y)**	
		**I6:** Strategies to deal with confounding factors were reported. Participants were grouped according to mathematics qualifications and sign-language experience, and these factors were incorporated into the statistical analyses. **(Y)**	
		**I7**: Outcomes were measured in a valid and reliable way for all participants. Standardized presentation procedures were used for all participants, and the rating scales demonstrated high reliability coefficients. **(Y)**	
		**I8:** Appropriate statistical analyses were used, including ANOVA, t-tests, correlation analyses, and regression models examining predictors of geometry-term recall. **(Y)**	
Ross B. M. (1975) [20]	JBI Analytical Cross‑Sectional	**I1:** Inclusion criteria were sufficiently described through the selection of deaf and hearing adolescent groups and age ranges. **(Y)**	Moderate methodological concerns
		**I2:** Participants, demographic characteristics, schools, sex distribution, and age ranges were reported in detail. **(Y)**	
		**I3:** Hearing status (deaf versus hearing) was clearly defined through recruitment from schools for the deaf and hearing high schools. **(Y)**	
		**I4:** Probability tasks and procedures were described extensively; however, formal evidence of validity and reliability of the measurement instruments was not reported**. (U)**	
		**I5:** Potential confounding factors such as age, educational experience, language deprivation, and mathematical training were acknowledged and discussed. **(Y)**	
		**I6:** No explicit statistical or methodological strategies were implemented to control for confounding variables**. (N)**	
		**I7**: Outcomes were measured using the same procedures and probability tasks across comparison groups. **(Y)**	
		**I8:** Appropriate statistical analyses were used, including chi-square tests and group comparisons. **(Y)**	
Palma et al. (2010) [21]	JBI Analytical Cross‑Sectional	**I1:** Inclusion criteria were partially reported; children with profound hearing loss treated with unilateral cochlear implantation were described, but eligibility criteria were not fully detailed. **(U)**	Moderate methodological concerns
		**I2:** Participants, age range, sex distribution, setting, and sample characteristics were described in detail. **(Y)**	
		**I3:** Hearing status was defined using explicit audiological criteria (profound hearing loss >90 dB HL and cochlear implantation). **(Y)**	
		**I4:** Numerical intelligence was assessed using the BIN 4–6 scales and cognitive abilities using Leiter‑R scales; however, formal validity and reliability data were not reported in the study. **(U)**	
		**I5**: Potential confounding factors, including intelligence quotient and rehabilitation duration, were identified and examined. **(Y)**	
		**I6:** Strategies to address confounding factors were reported through linear regression analysis assessing the influence of IQ and rehabilitation duration on counting performance. **(Y)**	
		**I7**: Outcomes were measured consistently across all participants using the same assessment protocol. (**Y**)	
		**I8:** Appropriate statistical analyses were used, including median tests and linear regression analyses. **(Y)**	
Kritzer KL. (2009) [22]	JBI Analytical Cross‑Sectional	**I1:** Inclusion criteria were clearly defined (children aged 4–6 years, deaf, no additional disabilities, not yet in first grade, ASL or spoken English at home). **(Y)**	Moderate methodological concerns
		**I2:** Participant characteristics, age, hearing status, family background, language exposure, assistive device use, and recruitment details were described. **(Y)**	
		**I3:** Hearing status and eligibility criteria were clearly defined. **(Y)**	
		**I4:** The TEMA-3 is a standardized instrument with reported reliability (internal consistency > .92). However, administration was modified and translated into ASL, which may affect measurement validity. **(U)**	
		**I5:** Potential confounding factors such as language exposure, parental hearing status, assistive listening devices, and home environment were identified and discussed. **(Y)**	
		**I6:** No specific analytical strategies were implemented to control for confounding factors. **(N)**	
		**I7**: Outcomes were measured consistently for all participants using the same assessment procedures. **(Y)**	
		**I8:** Descriptive and comparative analyses were appropriate for the study aims, although the analyses remained relatively simple and exploratory. **(Y)**	
Pagliaro CM (2013) [23]	JBI Analytical Cross‑Sectional	**I1:** Inclusion criteria were clearly defined (children aged 3–5 years with hearing loss enrolled in educational services; recruitment procedures described). **(Y)**	Moderate methodological concerns
		**I2:** Participant characteristics, age, gender, hearing level, assistive devices, communication mode, parental education and socioeconomic background were reported in detail. **(Y)**	
		**I3:** Hearing status was clearly defined through audiological classification (mild–profound hearing loss, cochlear implants, hearing aids, unilateral loss). **(Y)**	
		**I4:** The TEMA-3 is a standardized instrument with reported reliability (> 0.92). However, the Performance-Based Tasks were researcher-developed and had not been formally tested for reliability and validity**. (U)**	
		**I5:** Potential confounding factors (SES, communication mode, disabilities, parental education, language exposure) were identified and discussed. **(Y)**	
		**I6:** No specific strategies were reported to control or adjust for confounding variables. **(N)**	
		**I7**: Outcomes were measured consistently across all participants using the same assessment procedures. **(Y)**	
		**I8:** Appropriate descriptive analyses were used to address the study aims and present performance patterns across domains. **(Y)**	
Swanwick R (2005) [24]	JBI Analytical Cross‑Sectional	**I1:** Inclusion criteria were sufficiently described. Test papers were selected from deaf pupils who completed the 2002 KS3 National Mathematics Tests, and sampling procedures were reported. **(Y)**	Moderate methodological concerns
		**I2:** Characteristics of the sample, educational settings, test tiers, and distribution of pupils were described. **(Y)**	
		**I3**: Deaf status was clearly identified through participation of deaf pupils from schools and specialist units. **(Y)**	
		**I4:** Outcomes were derived from the UK National Curriculum Mathematics Tests and an established QCA coding framework; however, no specific reliability data for the coding procedure within this study were reported. **(U)**	
		**I5:** Potential confounding factors such as school placement, communication approach, curriculum access, language skills, and teaching experience were identified and discussed. **(Y)**	
		**I6:** No statistical or methodological strategies were employed to control confounding factors. **(N)**	
		**I7**: The same national examination and coding procedures were used for all papers analysed. **(Y)**	
		**I8:** Appropriate descriptive comparative analyses were conducted between the deaf sample and national hearing data. **(Y)**	
Kritzer KL (2009) [25]	JBI Checklist for Qualitative Research	**I1:** Congruity between stated philosophical perspective and methodology was not explicitly stated. **(U)**	Moderate methodological concerns
		**I2:** Congruity between methodology and research question/objectives. The study aimed to explore opportunities for mathematical learning in natural settings and used qualitative case-study methods. **(Y)**	
		**I3:** Congruity between methodology and data collection methods (full-day observations, field notes). **(Y)**	
		**I4:** Congruity between methodology and data representation/ analysis (coding, thematic analysis). **(Y)**	
		**I5:** Congruity between methodology and interpretation of results. **(Y)**	
		**I6:** Researcher positioning/reflexivity was not adequately addressed. **(N)**	
		**I7**: Influence of the researcher on the research, and vice versa, was not discussed. **(N)**	
		**I8:** Participants and their voices were adequately represented through detailed observations and direct interaction examples. **(Y)**	
		**I9:** Ethical aspects were not clearly reported in the article. **(U)**	
		**I10:** Conclusions were supported by the data and illustrative examples from observations. **(Y)**	
Laria C. et al., (2025) [26]	JBI Case-Control Checklist	**I1:** Cases and controls were recruited using clearly defined inclusion and exclusion criteria. **(Y)**	Moderate methodological concerns
		**I2:** Participants, age, study setting, and sample characteristics were described in detail. **(Y)**	
		**I3:** Hearing loss status was identified using standardized audiological assessments and explicit diagnostic criteria. **(Y)**	
		**I4:** Mathematical performance was assessed using the standardized BDE-2 battery; the instrument was described in detail and reported as validated. **(Y)**	
		**I5:** Potential confounding factors, including language abilities, working memory, and executive functions, were identified and discussed. **(Y)**	
		**I6:** No specific strategies were reported to control for confounding factors. **(N)**	
		**I7**: Mathematical outcomes were assessed consistently across cases and controls using the same procedures. **(Y)**	
		**I8:** The exposure (hearing loss) was measured uniformly using standardized audiological procedures in all participants. **(Y)**	
		**I9:** Hearing loss was an established condition and the exposure period was sufficient to evaluate its association with mathematical performance. **(Y)**	
		**I10:** Appropriate statistical analyses were used, including t-tests, Mann–Whitney tests, Wilcoxon tests, Fisher’s exact tests, and Spearman correlation analyses. **(Y)**	

Overall, the methodological rigor of the included studies was heterogeneous. Most studies fulfilled several applicable JBI criteria, particularly those related to the clear description of participants and study settings, the use of clearly defined outcome measures, and the application of appropriate statistical analyses. However, methodological limitations were identified across the body of evidence.

The most frequently observed concerns included small sample sizes, limited consideration or management of potential confounding factors, and incomplete reporting of methodological procedures. In several studies, confounding variables were insufficiently addressed or not explicitly controlled for, thereby reducing confidence in the interpretation of group differences. Small sample sizes were particularly common among studies involving deaf and hard-of-hearing participants and may have limited statistical power and generalizability.

Additional limitations included incomplete descriptions of participant selection procedures, insufficient information regarding the validity or reliability of some assessment measures, and restricted reporting of methodological details. Furthermore, studies published in earlier decades reflected reporting standards that were less comprehensive than those expected in contemporary research, making the assessment of some methodological domains more challenging.

Despite these limitations, many studies demonstrated important methodological strengths, including clearly defined participant groups, standardized assessment procedures, appropriate comparative designs, and transparent reporting of analytical methods. The detailed presentation of individual JBI appraisal items in Table 3 allows transparent evaluation of the methodological strengths and limitations of each study and facilitates interpretation of findings within the context of the overall evidence base.

No studies were excluded on the basis of methodological appraisal alone. Instead, the results of the quality assessment were considered during the interpretation and synthesis of findings, allowing potential sources of bias and methodological limitations to be taken into account when evaluating the overall body of evidence.

Table 3 summarizes the methodological appraisal of the included studies, including the JBI appraisal tool applied, item-level ratings for each checklist domain, and the overall methodological appraisal assigned through qualitative evaluation of study strengths and limitations.

As reported in Table 3, the methodological quality of the included studies was generally characterized by moderate methodological concerns. Most studies demonstrated methodological strengths in several JBI appraisal domains, particularly in the description of participants and study settings, the definition of hearing status, the use of consistent outcome assessment procedures, and the application of appropriate statistical analyses.

Across the included studies, the most frequently identified methodological limitation concerned the management of potential confounding factors. Although many authors acknowledged variables that could influence mathematical performance, explicit strategies to control or adjust for these factors were often absent. In addition, several studies provided limited information regarding the validity and reliability of the assessment tools used, resulting in *unclear* ratings for this domain.

Two studies, conducted by Rodríguez-Santos et al. [17] and Lang et al. [19], were judged to present low methodological concerns, reflecting clear participant selection criteria, appropriate consideration of potential confounding variables, consistent outcome measurement procedures, and robust statistical analyses.

The remaining ten studies were classified as presenting moderate methodological concerns. These concerns were mainly related to limited control of confounding variables, incomplete reporting of measurement validity or reliability, and small sample sizes, which may reduce the generalizability of findings.

No studies were classified as presenting high methodological concerns. Accordingly, no studies were excluded on the basis of methodological quality. Instead, the results of the methodological appraisal were considered during the interpretation and synthesis of the evidence, allowing potential sources of bias and study limitations to be taken into account when evaluating the overall findings of the review.

## Discussion

The present systematic review synthesized evidence from twelve observational studies examining mathematical competencies in deaf and hard of hearing (DHH) individuals under the age of 18 [15–26]. Taken together, these studies suggest a heterogeneous and domain-specific profile of mathematical performance, rather than a generalized impairment. Given the substantial heterogeneity across included studies, findings were interpreted by considering key characteristics, including age group, type of mathematical task, communication and educational context. Methodological quality was systematically considered in the interpretation of findings, with greater emphasis given to results from studies presenting low methodological concerns, while findings from studies presenting moderate methodological concerns were interpreted more cautiously and primarily used to provide contextual or supporting information. Importantly, the consistency of these patterns must be interpreted in light of the methodological appraisal of the included studies, which was predominantly characterized by moderate methodological concerns, with only two studies presenting low methodological concerns.

When considering task characteristics, difficulties appeared to be more pronounced in mathematically demanding tasks with high linguistic components. Across studies with generally moderate methodological concerns and relatively consistent findings [16–19,22–25], converging evidence suggests that mathematical difficulties in DHH learners are more pronounced in domains requiring conceptual understanding and linguistic mediation. Titus [15], rated as moderate methodological concerns, reported specific difficulties in the conceptual understanding of fractions despite clearly defined outcomes. These findings were echoed in earlier work by Ross and Hoemann [20], a comparative observational study—although presenting moderate methodological concerns and additional limitations related to outdated procedures and reporting standards—which suggested a developmental lag in complex probability reasoning rather than a complete absence of formal operational thinking. However, given their methodological limitations, older studies were interpreted cautiously and did not substantially influence the overall conclusions, which are primarily based on more recent and methodologically robust evidence.

When stratified by age, studies in preschool populations generally highlighted early emerging differences in language-mediated mathematical tasks.

Early mathematical development emerges as a particularly critical period for the onset of these differences. Evidence from recent observational studies in preschool populations supports the presence of early emerging gaps. Kritzer [22], using a standardized measure of early numeracy (TEMA-3), showed that most deaf children aged 4–6 years performed below age expectations, particularly in tasks requiring language-mediated reasoning such as word problems and number comparisons. Similarly, Pagliaro and Kritzer [23], in a cross-sectional baseline assessment of preschool-aged DHH children, showed that weaknesses are not confined to number skills but extend across multiple domains, including problem solving and measurement, while some areas such as geometry appear relatively preserved. Together, these findings further support this interpretation.

Evidence from studies with fewer methodological concerns provided additional support for domain-specific interpretations. Zarfaty, Nunes, and Bryant [16], rated as presenting moderate methodological concerns, showed that young deaf children performed comparably to hearing peers on spatial number tasks but showed weaknesses in temporal numerical processing, a finding derived from well-controlled task-based assessments despite a small sample. Rodríguez Santos et al. [17], rated as presenting low methodological concerns, showed intact symbolic and nonsymbolic magnitude representations in DHH children, with slower response times only in symbolic tasks, suggesting delayed access to numerical symbols rather than deficits in quantity representation.

More recent evidence from a clinically based case–control study further corroborates this interpretation. Laria et al. [26], rated as presenting moderate methodological concerns, applied a standardized dyscalculia-oriented battery (BDE-2) to school-aged children with hearing loss and normal hearing peers. While basic numerical recognition and number sense were relatively preserved, children with hearing loss showed significantly lower performance in calculation and overall mathematical indices. Importantly, no association emerged between the degree of hearing loss and mathematical outcomes, supporting the notion that symbolic and linguistically mediated components—rather than core numerical representations—are primarily implicated in mathematical difficulties among DHH learners.

Findings from larger observational studies also support this interpretation. Blatto-Vallee et al. [18], a relatively large observational study supported by comprehensive statistical analyses, showed that deaf students’ mathematical problem-solving performance was closely related to the type of visual–spatial representations used. The robustness of this evidence is strengthened by the large sample size and comprehensive statistical analyses, indicating that representation quality—rather than sensory status alone—plays a key role in mathematical outcomes.

Contextual and linguistic factors emerged most clearly in studies explicitly addressing language mediation. Kritzer’s qualitative observational study [25] highlighted the role of parental mediation in early numeracy development, showing that mathematically rich interactions supported deeper engagement with numerical concepts, although the findings should be interpreted in light of the methodological limitations identified in the quality appraisal. Lang and Pagliaro [19], rated as presenting low methodological concerns, further showed that imagery and familiarity of mathematical terms predicted recall performance in deaf high school students, underscoring the influence of linguistic representation on mathematics learning. Complementing this evidence, Swanwick et al. [24] showed that school-aged deaf students performed more poorly than hearing peers particularly in tasks requiring interpretation of linguistically complex mathematical information, while showing relatively better performance in routine computational tasks. This reinforces the central role of language accessibility in shaping mathematical outcomes.

Evidence from clinical observational research adds an additional layer of complexity. Palma et al. [21], rated as presenting moderate methodological concerns, reported lower numerical intelligence in preschool children with cochlear implants, with numerical performance closely associated with fluid reasoning and overall cognitive functioning. Although constrained by very small sample size, these findings suggest that early numerical difficulties may reflect broader interactions between auditory access, cognitive development, and representational skills.

Although a formal subgroup analysis was not feasible due to methodological heterogeneity, this structured interpretation provides a consistent framework for understanding variability across studies.

From a methodological standpoint, the variability in study quality reflects both historical differences in research standards and structural challenges inherent in studying low-incidence populations. While older studies and studies presenting moderate methodological concerns warrant cautious interpretation [20], the convergence of findings across studies presenting low and moderate methodological concerns [16–19,22–25] supports the interpretation that mathematical performance in DHH learners is not uniformly impaired and is consistently associated with linguistic accessibility, task demands, and representational strategies.

The predominance of studies presenting moderate methodological concerns suggests that the available evidence should be interpreted cautiously, particularly with regard to the influence of potentially uncontrolled confounding factors.

These results highlight the importance of language-mediated conceptual processing and educational context. Importantly, this pattern differs from what is typically described in developmental dyscalculia, where core deficits in numerical processing are more prominent.

This perspective has clear implications for assessment and instruction, emphasizing the need to disentangle linguistic demands from core numerical competencies when evaluating mathematical abilities in DHH children and adolescents.

## Conclusion

This systematic review synthesized evidence from twelve observational studies examining mathematical competencies in deaf and hard of hearing (DHH) individuals during childhood and adolescence. Overall, the findings indicate that mathematical performance in DHH learners is heterogeneous and not characterized by a generalized deficit. Instead, difficulties tend to be domain-specific and are more pronounced in mathematical tasks that place substantial demands on language processing and symbolic reasoning, such as calculation, measurement, and higher-level problem solving. In contrast, visually supported and non-symbolic components of mathematics, including basic number sense and some aspects of spatial processing, often appear relatively preserved.

Across the reviewed studies, linguistic factors—particularly access to a fully accessible language and overall language proficiency—emerged as key variables associated with mathematical outcomes. Evidence from both preschool and school-aged populations further suggests that these differences may originate early in development and remain observable across childhood and adolescence. These findings suggest a close association between mathematical performance and language development in DHH individuals, rather than being directly determined by hearing status or the severity of hearing loss alone. Educational context, communication modality, age of language acquisition, and hearing-related characteristics further contribute to the large interindividual variability observed.

From a methodological perspective, most included studies presented moderate methodological concerns, primarily related to small sample sizes, heterogeneous participant characteristics, limited control of confounding variables, and incomplete reporting of measurement validity and reliability. The use of assessment instruments not specifically validated for DHH populations and the variability in task design further constrain comparability across studies. These limitations highlight the need for further methodologically rigorous research, including clearer descriptions of linguistic and cognitive profiles and standardized assessments targeting distinct mathematical domains. Future research should aim to further disentangle linguistic and numerical components of mathematical performance, particularly from early childhood, and to inform the development of targeted, evidence-based educational and clinical interventions for DHH learners.

In conclusion, mathematical difficulties in DHH individuals should be understood within a broader linguistic and educational framework. Assessment practices and instructional approaches should explicitly account for language accessibility and task-specific demands.

These findings should be interpreted with caution, given the limited number of included studies and the predominance of studies presenting moderate methodological concerns. Overall, the available evidence remains limited and heterogeneous, and the conclusions should therefore be considered indicative rather than definitive.

### Limitations

This review has some limitations that should be considered when interpreting the findings. First, although the review aimed to identify the available studies in the literature addressing mathematical competencies in deaf and hard of hearing (DHH) children under the age of 18, the overall number of eligible studies was limited. This primarily reflects the current state of the evidence base, although the use of strict eligibility criteria may also have contributed to the limited number of included studies. Nevertheless, it highlights the need for further research in this population.

Second, the included studies showed substantial heterogeneity in terms of sample characteristics, assessment tools, mathematical domains examined, and reporting practices. This variability limited direct comparability across studies and precluded the possibility of conducting a quantitative synthesis. In particular, mathematical performance was assessed using a wide range of non-comparable instruments (e.g., standardized tests, task-based experimental measures, and qualitative assessments), and effect size data were not consistently reported across studies. In addition, the use of strict inclusion criteria may have led to the exclusion of potentially relevant studies, although this choice was made to ensure greater methodological homogeneity and interpretability of the findings.

Furthermore, several studies were characterized by small sample sizes and incomplete reporting of relevant confounding variables, such as linguistic background, socioeconomic status, and educational context. These factors may have contributed to variability in the reported outcomes and constrained the generalizability of individual findings. Given the observational nature of the included studies, the findings should be interpreted as evidence of associations rather than causal relationships. Therefore, it is not possible to determine whether hearing status, language accessibility, educational context, or other related factors directly influence mathematical performance in DHH children and adolescents. Finally, some older studies did not fully meet contemporary standards of methodological transparency, which influenced quality ratings but also reflects historical differences in research practices.

The search strategy was conducted using PubMed, Scopus, and Web of Science, which provide broad multidisciplinary coverage of biomedical, clinical, and educational research. However, additional databases specifically focused on education and psychology, such as ERIC and PsycINFO, were not searched. Consequently, some potentially relevant studies may not have been identified and this should be considered when interpreting the completeness of the evidence base. In addition, the search strategy did not include grey literature sources, which may have limited the identification of some potentially relevant studies.

Despite these limitations, this review provides a structured overview of the available evidence and identifies consistent patterns that can inform future research and educational practice. The methodological appraisal indicated that most included studies presented moderate methodological concerns, particularly regarding the management of confounding factors and the reporting of measurement validity and reliability. These limitations should be considered when interpreting the overall strength of the evidence.

## Supporting information

S1 Checklist PRISMA 2020 Checklist. (DOCX)

S1 Dataset Extracted dataset used for study selection, methodological appraisal, and narrative synthesis. (XLSX)
