# Supplementary material for: Mathematical skills in deaf and hard-of-hearing children: A systematic review
Source: PLoS One. 2026 Sep 16;21(9):e0358324. doi: 10.1371/journal.pone.0358324 (PMC13581004; doi:10.1371/journal.pone.0358324)
Supplement: Supplementary file 2 [file pone.0358324.s003.docx]

# **Peer Review Report**

# **Journal:** PLOS One

**Manuscript ID:** PONE-D-26-21292

# **Title:** Mathematical Skills in Deaf Patients: A Systematic Review

# **Recommendation:** Major Revision

This systematic review addresses an important educational and clinical topic concerning mathematical skills among deaf and hard-of-hearing (DHH) children and adolescents. The manuscript is generally well organized and follows a PRISMA-oriented structure. However, several methodological and reporting issues limit the transparency, reproducibility, and interpretability of the findings. In its current form, the manuscript requires substantial revision before it can be considered for publication.

**Reviewer’s Evaluation**

### **General Comments**

The manuscript addresses an important topic and generally follows an appropriate systematic review methodology. However, I have several concerns regarding its technical robustness. First, there are inconsistencies in reporting the number of included studies throughout the manuscript, which raises concerns about the transparency of the study selection process. Second, the methodological quality assessment using the JBI critical appraisal tools requires further justification, particularly regarding the conversion of checklist outcomes into overall quality categories and risk-of-bias classifications. Third, substantial heterogeneity exists across the included studies in terms of participant characteristics, mathematical outcomes, and assessment methods, limiting the strength of the conclusions that can be drawn. Finally, some conclusions appear stronger than warranted by the available evidence, especially given the small number of included studies and the methodological limitations identified in several of them. Therefore, while the review is potentially valuable, major revisions are needed before I can conclude that the data fully support the authors’ conclusions.

**Major Comments**

1. Although the authors report the databases searched and provide general search strings, the search methodology lacks sufficient detail for full reproducibility. Specifically:

- The exact search dates should be reported consistently throughout the manuscript.
- It is unclear whether database-specific adaptations of the search strategy were implemented.
- No information is provided regarding the use of filters, language restrictions, document-type restrictions, or manual reference screening.
- The authors should provide complete reproducible search strategies for each database, preferably as supplementary material.

Because systematic reviews rely heavily on reproducible search procedures, additional detail is necessary.

2. There are several inconsistencies throughout the manuscript regarding the number and classification of included studies. For example:

- The Results section reports that eight studies met the eligibility criteria, whereas later sections repeatedly state that nine studies were included.
- The PRISMA flow description should be carefully revised to ensure consistency across the text, figure, and tables.
- The numerical accounting of excluded studies at different screening stages should be verified because some totals do not appear to reconcile clearly.

These discrepancies raise concerns regarding the accuracy of the study selection process.

3. The rationale for excluding intervention studies, randomized trials, and clinical trials is not adequately explained. Since intervention studies may provide valuable information regarding mathematical performance in DHH populations, the authors should better justify their exclusion and explain how this decision aligns with the review objective. Similarly, the restriction to observational studies should be more explicitly justified.
4. The use of JBI critical appraisal tools is appropriate; however, the approach used to derive overall quality ratings is insufficiently described. Several concerns arise:

- The manuscript converts JBI checklist scores into categorical quality levels without providing references supporting this scoring procedure.
- JBI tools were not originally designed to generate numerical quality scores.
- The rationale for the selected cut-off values (≥70%, 50–69%, <50%) should be justified.
- The process used to determine overall risk-of-bias judgments remains unclear.

The authors should provide a stronger methodological justification or reconsider how quality assessments are summarized.

5. The Discussion repeatedly emphasizes methodological quality categories when interpreting study findings. However, no structured sensitivity analysis or evidence synthesis framework was used. The manuscript would benefit from a clearer explanation of how study quality influenced interpretation of the evidence and conclusions.
6. Although the authors evaluate methodological quality using JBI tools, no assessment of the overall certainty or strength of evidence is provided. While not mandatory, the inclusion of a framework such as GRADE or a justified alternative approach would substantially strengthen the interpretation of the findings and improve transparency regarding confidence in the conclusions.
7. The review includes only nine heterogeneous studies, several with small sample sizes and moderate methodological limitations. Therefore, statements suggesting that mathematical difficulties in DHH learners are primarily explained by linguistic accessibility should be presented more cautiously. The conclusions should better acknowledge the limited evidence base and avoid implying causal relationships that cannot be established from the included observational studies.

## **Minor Comments**

1. The manuscript title uses the term **"Deaf Patients"**, whereas most included studies concern children and students in educational contexts rather than clinical patients. The authors should consider replacing the term "patients" with a more appropriate population descriptor such as: **"Mathematical Skills in Deaf and Hard-of-Hearing Children and Adolescents: A Systematic Review".**
2. The manuscript is generally understandable; however, several sections would benefit from careful English-language editing. Examples include: grammatical inconsistencies, punctuation issues, occasional typographical errors, repeated wording. A professional language revision is recommended.
3. The authors state that PRISMA 2020 guidelines were followed. However, several PRISMA items require further clarification, including: protocol registration details, screening procedures, handling of disagreements, assessment of reporting bias, justification for synthesis methods. The authors should ensure complete compliance with the PRISMA 2020 checklist.
4. Tables 2 and 3 are informative but would benefit from improved formatting and standardization. Several study descriptions are overly detailed, whereas others provide insufficient information regarding sample characteristics and outcome measures.
