# Supplementary material for: Mathematical skills in deaf and hard-of-hearing children: A systematic review
Source: PLoS One. 2026 Sep 16;21(9):e0358324. doi: 10.1371/journal.pone.0358324 (PMC13581004; doi:10.1371/journal.pone.0358324)
Supplement: Supplementary file 3 [file pone.0358324.s004.docx]

**Review Comments to the Author**

**Reviewer #1:**

**Summary:**

This manuscript is a literature review of studies conducted to examine the mathematics performance of Deaf children under the age of 18 years of age. Using a highly restrictive set of review criteria, the body of mathematics research in the field was reduced to a set of 9 studies. A review of these studies indicates that hearing loss itself is not a cause of low mathematics performance, but rather the low language and educational situations that may accompany the hearing loss.

**Strengths:**
Mathematics is an under-researched area of study in Deaf Education so research that focuses on this area is much needed. The manuscript is interesting and well written.

[Reply]: We thank the Reviewer for these positive comments and for highlighting the relevance of the topic.

We agree that mathematics in deaf and hard-of-hearing (DHH) populations remains a relatively under-investigated area within Deaf Education, and one of the motivations of this review was to contribute to addressing this gap in the literature.

We also appreciate the Reviewer’s acknowledgment of the clarity and readability of the manuscript.

**Areas of Concern:**
• The reference to the Deaf individuals under focus in this manuscript as “patients” in the title is concerning to this reviewer. In every study reviewed in this manuscript, the participants were children. The children were students at a school, or members of a family- they were not sick, and they were not in a hospital. Please find a new word.

[Reply]: Thank you for your suggestion. Accordingly, we have revised the title to replace the term “patients” with “children”, in order to better reflect the characteristics and context of the populations included in the review. This change has also been applied throughout the manuscript where relevant, to ensure consistency and to avoid unintended medicalization of the study population.

• By using such highly restrictive criteria, many good studies were eliminated from review that might have provided good information.

[Reply]: Thank you for this insightful comment. We acknowledge that the use of restrictive inclusion criteria may have led to the exclusion of potentially relevant studies.

However, the inclusion criteria were intentionally defined a priori, in accordance with the PICOS framework and PRISMA 2020 guidelines, to ensure methodological consistency and comparability across studies. In particular, we limited the review to observational studies involving participants under 18 years of age and requiring direct assessment of mathematical skills. This approach was chosen to specifically investigate the relationship between hearing status and mathematical performance in naturalistic contexts, without the confounding influence of intervention effects or indirect outcome measures.

To address the reviewer’s concern and improve clarity, we have made the following revisions to the manuscript:

- We clarified the observational nature of the included studies in the Abstract (Methods section), explicitly stating that this is a systematic review of observational studies.

- We refined the study aim in the Introduction by specifying “observational methodological approaches,” ensuring full alignment between the stated objective and the inclusion criteria.

- We added a statement at the end of the Inclusion Criteria section to explicitly justify the rationale for adopting stricter selection criteria in terms of methodological consistency, internal validity, and interpretability of findings.

- We further acknowledged this aspect in the Limitations section, noting that strict inclusion criteria may have resulted in the exclusion of potentially relevant studies, while emphasizing that this decision was made to enhance methodological homogeneity.

While this approach may have reduced the number of eligible studies, it allowed for a more focused and coherent synthesis of the available evidence.

• The authors state in the conclusion that “These limitations highlight the need for more robust and methodologically rigorous research, including clearer descriptions of linguistic and cognitive profiles and standardized assessments targeting distinct mathematical domains.” While this reviewer does not doubt the validity of this statement, it is important to remember the characteristics of the population that one is working with. Deaf children are a very heterogenous population. Cognitive profiles are dependent on language, and these are often difficult to determine when children are young. Then, in using standardized assessments, whom should these assessments be standardized on? Hearing children? Tests standardized for hearing children are often not appropriate for deaf children, and tests created for Deaf children are difficult to develop because of the heterogeneity of the population. The challenges to creating rigorous research are numerous in Deaf Education. Nevertheless, there is at least one study out there, that this reviewer knows of, that did use a standardized assessment- for a population in the appropriate age range that was not included in the literature review for this manuscript. Not sure why.

[Reply]: We thank the reviewer for this thoughtful and insightful comment. We fully agree that deaf and hard of hearing (DHH) children represent a highly heterogeneous population, and that linguistic access plays a critical role in shaping cognitive and mathematical development, particularly in early childhood. We also acknowledge the inherent challenges in applying standardized assessments in this population, given that most available tools are normed on hearing children, while instruments specifically designed for DHH learners are difficult to develop due to the variability in language exposure and communication modalities.

In response to this comment, we have revised the Conclusion section to better reflect these methodological and conceptual challenges, emphasizing that mathematical performance in DHH individuals must be interpreted within a broader linguistic and educational framework, rather than relying solely on standardized assessment outcomes.

Regarding the reviewer’s observation about the use of standardized assessments, we agree that this is a critical issue. We have carefully re-examined the literature and identified additional relevant studies that employed standardized or structured assessments in DHH populations, including Kritzer (2009), Pagliaro and Kritzer (2013), and Swanwick et al. (2005). These studies have now been included in the review through a manual screening process and integrated into the analysis. Their inclusion further supports the conclusion that mathematical difficulties in DHH individuals are closely linked to language-mediated processes and task demands, rather than representing a uniform deficit.

We appreciate the reviewer’s suggestion, which has strengthened both the comprehensiveness and the methodological rigor of the manuscript.

____________________________________________________________________

**Reviewer #2:**

The topic is highly relevant to the fields of mathematics education, special education, and cognitive development. Overall, the study addresses an important gap in synthesizing evidence on domain-specific mathematical abilities and the role of linguistic access in DHH learners.

**Major strengths**

The review demonstrates several strengths. First, it follows PRISMA 2020 guidelines and uses a predefined protocol registered on OSF, which enhances transparency and reproducibility. Second, the search strategy is comprehensive, covering major databases (PubMed, Scopus, Web of Science) and employing both controlled vocabulary and free-text terms. Third, the inclusion of a structured quality appraisal using JBI tools adds methodological rigor and allows for a nuanced interpretation of study quality. Finally, the narrative synthesis is well-developed and appropriately highlights the heterogeneity of findings, particularly the distinction between linguistic and non-linguistic mathematical tasks.

[Reply]: We thank Reviewer for the positive evaluation of our manuscript and for highlighting the relevance of the topic and the strengths of the methodology and synthesis.

**Major concerns**

Despite these strengths, several methodological and reporting issues require attention:

**Inconsistencies in study selection reporting**
There is inconsistency in the number of included studies. The manuscript reports both eight and nine included studies in different sections. Additionally, the PRISMA flow reporting contains numerical inconsistencies (e.g., 579 records minus exclusions does not consistently lead to the reported final sample). These discrepancies should be carefully verified and corrected.

[Reply]: Thank you for your comment. We rechecked the study selection process and corrected the numerical inconsistencies throughout the manuscript. The final number of included studies is 12. We also revised the PRISMA flow diagram and the Results section to ensure consistent reporting of all records identified, screened, excluded, and included.

**Clarification of eligibility criteria**
The inclusion/exclusion criteria are detailed but somewhat repetitive and occasionally unclear (e.g., overlapping descriptions of study designs). A more concise and structured presentation of PICOS criteria would improve clarity.

[Reply]: Thank you for your comment. The eligibility criteria have been revised to improve clarity and reduce redundancy. In particular, overlapping descriptions of study designs have been removed, and the PICOS framework has been streamlined to provide a more concise and structured presentation.

**Lack of quantitative synthesis justification**
Although a meta-analysis is not required, the authors should more explicitly justify the absence of quantitative synthesis. Given that some included studies report comparable outcomes, a brief discussion of effect size heterogeneity or feasibility of meta-analysis would strengthen methodological transparency.

[Reply]: Thank you for your comment. A clarification has been added in the Limitations section to explicitly justify the absence of a quantitative synthesis. In particular, we now state that a meta-analysis was not feasible due to substantial heterogeneity across studies in terms of study design, outcome measures, and participant characteristics, as well as the lack of comparable effect size data.

**Quality appraisal interpretation**
While JBI tools are appropriately applied, the categorization of risk of bias (low/moderate/high) appears partially subjective and not fully standardized. A clearer explanation of thresholds and consistency across different JBI checklists is needed. Additionally, combining qualitative and quantitative studies under similar appraisal logic may require further justification.

[Reply]: Thank you for your comment. In response, the methodological quality assessment process was substantially revised. We removed the use of numerical thresholds and score-based categorizations, as these approaches are not recommended by the Joanna Briggs Institute. Methodological quality is now interpreted using a qualitative framework based on individual JBI appraisal items and key methodological domains rather than predefined cut-off values. In addition, Table 3 was completely redesigned to report the individual JBI items, their ratings (Yes/No/Unclear), and study-specific methodological notes supporting the appraisal process. This approach improves transparency, avoids the use of non-standard scoring systems, and provides a clearer and more detailed justification for the methodological appraisal of each included study.

**Heterogeneity of included studies**
The included studies vary substantially in design, sample size, age range, and outcome measures. Although this is acknowledged in the discussion, a more structured subgroup interpretation (e.g., by age group, task type, or communication modality) would improve analytical depth.

[Reply]: Thank you for your comment. The Discussion has been revised to provide a more structured interpretation of findings according to key study characteristics, including task type and age group. We also added a statement clarifying that a formal subgroup analysis was not feasible due to heterogeneity, while adopting a structured narrative approach to improve interpretability.

Particularly, we have added the following sentences in the Discussione section:

1) Given the substantial heterogeneity across included studies, findings were interpreted by considering key study characteristics, including age group, type of mathematical task, and communication and educational context.

2) When considering task characteristics, difficulties were more pronounced in mathematically demanding tasks with high linguistic components.

3) When stratified by age, studies in preschool populations consistently highlighted early emerging differences in language-mediated mathematical tasks.

4) Although a formal subgroup analysis was not feasible due to methodological heterogeneity, this structured interpretation provides a consistent framework for understanding variability across studies.

**Older studies and methodological limitations**
Several included studies are dated (e.g., 1975), and their methodological limitations are acknowledged. However, their influence on overall conclusions should be more critically weighted, as older methodologies may not be fully comparable to contemporary research standards.

[Reply]: Thank you for your insightful comment. We agree that older studies require careful consideration due to differences in reporting standards and methodological practices compared with contemporary research.

In our review, only two studies predate 2000 (1975 and 1995), and both were critically appraised using the JBI tools and interpreted in light of their methodological strengths and limitations. In particular, the study by Ross and Hoemann (1975) presented methodological limitations related to reporting standards, measurement validity reporting, and limited control of potential confounding factors. These limitations were explicitly considered during interpretation of the findings.

Importantly, this issue has been addressed in the revised manuscript. In the Discussion, we now emphasize that older studies were interpreted cautiously and primarily used to provide historical and contextual perspectives rather than to drive the overall conclusions. For example, we added the following statement:

*“However, given their methodological limitations, older studies were interpreted cautiously and did not substantially influence the overall conclusions, which are primarily based on more recent evidence.”*

We also clarified that the interpretation of the evidence considered the methodological appraisal of individual studies and the consistency of findings across the available literature. Accordingly, conclusions were based primarily on convergent findings across studies presenting generally moderate methodological concerns and relatively consistent results, rather than on isolated findings from older studies.

These revisions ensure a more balanced interpretation of the evidence and acknowledge the methodological limitations associated with earlier research while preserving their contextual value within the overall synthesis.

**Language and reporting clarity**
Minor grammatical inconsistencies and repetition appear throughout the manuscript (e.g., repeated phrases in Methods and Results sections). A careful language revision is recommended to improve readability and precision.
[Reply]: Thank you for your suggestion. We have carefully revised the manuscript to improve language clarity and readability. Minor grammatical inconsistencies have been corrected, redundant expressions have been reduced, and phrasing has been streamlined, particularly in the Methods and Results sections. In addition, all residual track changes artifacts have been removed to ensure consistency and precision in the final version

**Minor comments**
The PRISMA flow description should be aligned with the final included study count.
[Reply]: Thank you for your comment. We carefully checked and aligned the PRISMA flow description, figure, and corresponding text to ensure consistency with the final number of included currently studies (n = 12) throughout the manuscript.

Table formatting (especially Table 2) would benefit from standardization for readability.
[Reply]: Thank you for your comment. We have revised Table 2 to improve formatting and ensure consistent presentation across all entries. In particular, we standardized the structure, alignment, and terminology used within the table to enhance readability.

Some methodological terms (e.g., “moderate–high quality”) require clearer operational definitions.
[Reply]: Thank you for your comment. We have corrected

Keywords could be refined to better align with indexing databases.
[Reply]: Thank you for your suggestion. We have revised the Keywords section to improve alignment with commonly used indexing terms. In particular, we refined the terminology to enhance clarity, consistency, and discoverability in indexing databases.

**Ethical and publication considerations**
No major ethical concerns are identified, as the study is based on published literature. However, the authors should ensure full transparency regarding protocol adherence and clarify whether any deviations from the registered protocol occurred. Additionally, consistency in reporting is essential to avoid concerns regarding data handling or selective inclusion.
[Reply]: Thank you for your comment. We confirm that the review was conducted in accordance with the registered protocol. No major deviations from the predefined review protocol occurred following its formalization and registration. To enhance transparency, we have carefully revised the manuscript to ensure consistency in reporting across all sections and to align the description of the methodology and study selection process with the registered protocol.

Additionally in main text we added in the Methods / Study design section:

“The review was conducted in accordance with the registered protocol, and no major deviations were made during the study selection process.”

**Conclusion**
This systematic review addresses an important and underexplored area with clear relevance to education and developmental psychology. With revisions to improve consistency, methodological transparency, and reporting clarity, the manuscript has the potential to make a meaningful contribution to the field.

[Reply]: Thank you for your positive and constructive evaluation of our manuscript. We appreciate your recognition of the relevance of the topic and the potential contribution of this study to the field. We have carefully addressed all the suggested revisions to improve consistency, methodological transparency, and reporting clarity, and we believe the manuscript has been significantly strengthened as a result.

________________________________________________________________________

**Reviewer #3:**

**Introduction**
The manuscript entitled “Mathematical Skills in Deaf Patients: A Systematic Review” offers a valuable and timely contribution to the growing body of literature examining cognitive and educational outcomes among deaf individuals. By synthesizing existing research on mathematical performance within this population, the authors provide important insights into both the challenges and potential strengths associated with numerical cognition in deaf learners. The review is particularly commendable for its thorough methodology and its well-developed discussion section, which thoughtfully interprets findings and situates them within broader educational and cognitive frameworks.
Overall, the authors have demonstrated considerable effort in compiling and analyzing relevant studies, and their work has clear implications for both research and practice. However, despite these strengths, there remain several issues that should be addressed to further improve the clarity, rigor, and impact of the manuscript. These concerns are outlined below.

**Title & Abstract**
• The title is precise and reflect the content, nevertheless, using the term “Deaf Patients” does not match the focus. The review is about children in education. It would fit better to say “Deaf and hard-of-hearing children and adolescents” or “DHH learners.”

[Reply]: Thank you for your comment. We agree that the original terminology did not accurately reflect the focus of the manuscript. Accordingly, we revised the title by replacing “patients” with “deaf and hard-of-hearing children,” ensuring consistency with the population examined in the included studies. This modification has also been applied throughout the manuscript where relevant, to improve clarity and avoid unintended medicalization of the study population.

• The abstract is clear, but it is missing key details; Mainly, how many databases you searched, the time period for the study, and the reason for the use of narrative synthesis instead of a meta-analysis.

[Reply]: Thank you for your comment. We have revised the Abstract to include additional methodological details, specifically specifying the number of databases searched and the time period covered by the search strategy. We have also clarified that a narrative synthesis was conducted and briefly justified this choice based on the heterogeneity of the included studies, in line with PRISMA reporting recommendations.

• According to the criteria, the paper included only nine studies, and their quality varies. I think the researchers should be careful in presenting the results where it sounded stronger than they are.

[Reply]: Thank you for your comment. We agree that caution is required when interpreting findings derived from a limited number of studies presenting variable methodological quality. In response, we revised the Results, Discussion, and Conclusion sections to ensure a more balanced interpretation of the evidence. Statements that could be perceived as overly strong were moderated, and greater emphasis was placed on the methodological limitations of the included studies. In addition, the methodological quality assessment was substantially revised. Table 3 was redesigned to provide detailed item-level JBI appraisals and study-specific methodological considerations, allowing a more transparent evaluation of the strengths and limitations of the evidence base. We also clarified that the overall findings should be interpreted cautiously given the predominance of studies presenting moderate methodological concerns and the heterogeneity of the available evidence. This approach was adopted to avoid overgeneralization and to ensure that conclusions remain proportionate to the strength of the available data.

**Introduction**
• The reason for the study is clear. The link between math skills and daily life is easy to understand.

[Reply]: Thank you for your positive comment. We appreciate the recognition of the clarity of the study rationale and the relevance of the link between mathematical skills and everyday functioning, which represents a key motivation for conducting this review.

• The section on developmental dyscalculia gets quite a lot of space relative to its role in the review. Further explanation is needed clearly why it's included as a comparator — and why wasn’t this issue featured in the discussion.
[Reply]: Thank you for your comment. Developmental dyscalculia was included as a conceptual comparator to contextualize mathematical difficulties in DHH children within the broader framework of mathematical learning disorders. In response, we have streamlined the Introduction to clarify this role and reduced its emphasis by explicitly framing developmental dyscalculia as a conceptual reference. We have also integrated this perspective into the Discussion by highlighting how the mathematical profile observed in DHH learners differs from that typically described in developmental dyscalculia, thereby improving coherence across sections.

• The introduction would benefit from a sharper statement of what gap in the existing systematic evidence this review is actually filling. It's implied, but never said directly.
[Reply]: Thank you for this insightful comment. We agree that the research gap was not sufficiently stated explicitly. In response, we have revised the Introduction to include a clearer and more direct statement of the gap addressed by this review, emphasizing the lack of systematic synthesis specifically focused on domain-specific mathematical skills in DHH children and adolescents and their relationship with linguistic and cognitive factors. This revision improves the clarity of the study rationale and the positioning of the review within the existing literature.

**Methods**
• The protocol statement needs fixing. The link is broken and the format is unclear. Include the platform, number, date, and whether it was registered before screening. Remove the extra “ù” symbol before the Inclusion Criteria and Eligibility.
[Reply]: Thank you for your comment. We have revised the protocol statement in the Methods section to correct the link and improve clarity of reporting. Specifically, we have included the registration platform (OSF Registries), DOI, and date of registration. We also clarified the timing of the registration in relation to the review process. In addition, the formatting of the section has been corrected and the extraneous “ù” symbol before the Inclusion Criteria and Eligibility section has been removed.

• The research question — "What is the impact of deafness on the development of mathematical skills?" — is too broad and implies CAUSALITY that observational studies can't establish. Since your criteria limit the population to under 18 years, the question should ask about the association between DHH and mathematical skill development among DHH children and adolescents?" this might be more appropriate and safer about the study designs involved.
[Reply]: Thank you for this important comment. We agree that the original research question implied causality, which is not appropriate given the observational nature of the included studies. In response, we have revised the research question to reflect an associative framework and to align with the study design and eligibility criteria. The research question has been modified as follows: “What is the association between deafness and the development of mathematical skills in deaf and hard-of-hearing (DHH) children and adolescents?”

• The eligibility criteria need a few clarifications:
o Were both congenital and acquired hearing loss included?

[Reply]: Thank you for your comment. We confirm that both congenital and acquired hearing loss were already included in the eligibility criteria, as specified in the PICOS framework under the exposure definition.

o Was the severity of hearing loss a factor?
[Reply]: Thank you for your comment. Severity of hearing loss was not used as an inclusion or exclusion criterion in the study selection process. However, when reported in the included studies, it was considered as a relevant variable and is discussed as a potential source of variability in mathematical outcomes.

o The phrase "when applicable" for the comparator group is vague — were studies without a comparison group eligible or not?
[Reply]: Thank you for your comment. We agree that the phrase “when applicable” was unclear. In response, we have revised the comparator criterion to explicitly clarify that studies without a comparison group were also eligible, provided they included a direct assessment of mathematical skills in DHH participants. This modification improves the transparency and consistency of the inclusion criteria.

o The outcome criterion is broad; the researchers needed to be specific about which cognitive processes counted and how they relate to mathematical outcomes.
[Reply]: Thank you for your comment. We agree that the outcome criterion required further clarification. In response, we have revised the description to more explicitly specify the types of cognitive processes considered and to clarify that only those directly related to mathematical performance were included. This modification improves the transparency and precision of the inclusion criteria.

• The search strategy is well reported. But using terms like “cohort” or “cross-sectional” may have excluded studies. Either explain this choice or remove those filters Adding ERIC, PsycINFO, EBSCO, or Education Source would also strengthen the coverage for this field.
[Reply]: Thank you for your comment. We agree that the use of study design filters may influence the retrieval of articles. In our approach, these terms were intentionally included to increase the specificity of the search toward observational studies, in alignment with the predefined eligibility criteria. To improve transparency, we have clarified this methodological choice in the Methods section.

In addition, we have revised the Limitations section to acknowledge the scope of the search strategy. While the review was conducted using major multidisciplinary databases (PubMed, Scopus, and Web of Science), which ensure broad and comprehensive coverage of the literature, the inclusion of additional education-focused databases might have identified further records. However, it is unlikely that major relevant studies were missed.

• The study selection process is described well, but the data extraction process is barely mentioned. Readers need to know who extracted the data, whether it was done independently or checked by a second person, what form was used, and how disagreements were resolved. If inter-rater reliability was calculated, report it.
[Reply]: Thank you for your comment. We agree that the description of the data extraction process required further detail. In response, we have revised the Methods section to explicitly describe the data extraction procedure, including the number of reviewers involved, the use of a predefined data extraction form, and the process for resolving disagreements. This information has now been clearly integrated into the Study Selection and Data Extraction section to improve methodological transparency.

• The quality appraisal is mostly appropriate, nevertheless, it was mentioned that cross-sectional checklist has nine items but list eight, this inconsistency needs to be fixed. The phrase "moderate expertise" for the reviewers slightly undermines confidence in the process, use more appropriate words focusing mainly on the training rather than the level. Furthermore, Also clarify how disagreements were resolved before involving the third reviewer.
[Reply]: Thank you for this valuable and detailed comment.

We have carefully revised the *Methodological Quality Assessment* section to address the reported inconsistency regarding the number of items included in the JBI checklist for analytical cross-sectional studies. Specifically, the checklist has now been correctly described as comprising eight items, and all corresponding descriptions have been aligned accordingly to ensure internal consistency throughout the manuscript.

In addition, we agree that the expression “moderate expertise” may unintentionally undermine confidence in the appraisal process. This wording has been revised to more appropriately emphasize the reviewers’ training and experience in systematic review methodology and critical appraisal.

Finally, we have clarified the procedure used to resolve disagreements between reviewers. The manuscript now explicitly states that discrepancies were first discussed and resolved through consensus between the two reviewers, and that the third reviewer was consulted only when consensus could not be reached. This clarification improves the transparency and rigor of the study selection and data extraction processes.

These revisions have strengthened both the clarity and the methodological transparency of the quality appraisal process.

• Your quality scoring is not standard for JBI. Either justify it or say you created it. It is better to say “higher,” “moderate,” or “lower quality.”
[Reply]: Thank you for this important and constructive comment. We agree that numerical quality scoring and predefined thresholds are not part of the recommended use of the JBI critical appraisal tools.

In response to this concern, we substantially revised the methodological quality assessment process. Numerical thresholds and score-based categorizations were removed, and methodological quality is now interpreted through a qualitative framework based on individual JBI appraisal items and key methodological domains. In addition, Table 3 was completely redesigned to provide item-level reporting of the appraisal process, including individual Yes/No/Unclear ratings and study-specific methodological considerations supporting the appraisal of each study.

We also revised the terminology used throughout the manuscript to avoid implying the use of a standardized scoring system. Methodological quality is now discussed using a qualitative interpretative approach rather than numerical classifications. These revisions improve transparency, provide a clearer justification for methodological appraisal judgments, and align the quality assessment more closely with the intended use of the JBI critical appraisal tools.

• One qualitative study was included, but this is not in the criteria. If qualitative studies were allowed, say so. If not, explain why it was included.
[Reply]: Thank you for this important comment. We acknowledge that the inclusion of a qualitative study was not explicitly specified in the original description of the eligibility criteria, which may have led to a lack of clarity.

To address this issue, we have revised the *Inclusion Criteria and Eligibility* section to explicitly state that both quantitative and qualitative observational studies were considered eligible. In particular, we have added the following clarification under the study design criterion:
“Observational studies, including cross-sectional, cohort, and case-control designs. Both quantitative and qualitative observational studies were considered eligible, provided they examined mathematical skills or related processes in DHH children.”

This modification ensures full consistency between the eligibility criteria and the included studies, including the qualitative study by Kritzer (2009).

In addition, we have specified in the *Methodological Quality Assessment* section that design-specific JBI critical appraisal tools were applied according to study type, including the checklist for qualitative research.

These revisions improve the transparency and coherence of the methodological framework.

**Results — Screening & Flow Diagram**
• The numbers are inconsistent. You say eight studies in one place and nine in another. Use one number everywhere.
[Reply]: Thank you for your comment.

We carefully rechecked the study selection process and identified inconsistencies in the reporting of the number of included studies across different sections of the manuscript. These discrepancies have now been fully corrected.

The final number of included studies is twelve (n = 12), and this has been consistently updated throughout the manuscript, including the Results section, the PRISMA flow diagram, the Abstract, and all relevant text.

In addition, we have revised the description of the screening process to ensure that all reported numbers are internally consistent and clearly reflect each stage of study selection.

These revisions improve the accuracy and transparency of the reporting.

• The PRISMA diagram is useful. But do not mix duplicate removal with other exclusions. Also, “not retrieved” is confusing.
[Reply]: Thank you for this helpful comment.

We have carefully revised the PRISMA flow diagram and its corresponding description to improve clarity and ensure full alignment with PRISMA 2020 reporting standards, while preserving the original study selection process.

Specifically, we have clarified the initial phase by appropriately distinguishing duplicate removal from other types of exclusions and by using clearer and more standard terminology (e.g., “records removed as duplicates”). In addition, ambiguous wording such as “not retrieved” has been removed.

We have also revised the screening stage by replacing vague expressions (e.g., “does not match with our topic”) with more standard PRISMA-compliant phrasing (i.e., “records excluded”), while detailed reasons for exclusion are provided at the full-text assessment stage.

Finally, minor inconsistencies and labeling issues within the diagram have been corrected to ensure internal coherence across all stages of the flowchart.

These revisions enhance both the transparency and the methodological rigor of the study selection process.

• "Does not match with our topic" as the reason for excluding 500 records is too vague. Even a brief list of the main reasons at that stage — wrong population, wrong outcome, wrong design — would make the screening process more transparent and reproducible.
[Reply]: Thank you for this important comment.

We agree that the expression “does not match with our topic” was too vague and did not provide sufficient transparency regarding the reasons for exclusion at the screening stage.

To address this, we have revised the PRISMA flow diagram by replacing this wording with the more standard and PRISMA-compliant expression “records excluded.” In addition, we ensured that detailed reasons for exclusion are clearly reported at the full-text eligibility stage, where categories such as wrong population, absence of direct mathematical outcomes, study design, and other specific criteria are explicitly listed.

This approach improves the transparency and reproducibility of the study selection process while maintaining clarity and consistency with PRISMA 2020 reporting recommendations.

**Table 2 — Study Descriptions**
• Several studies are listed without exact sample sizes. Try to include numbers for all. If estimated please mention it. Leaving it blank makes it very hard to assess the weight of evidence.
[Reply]: Thank you for your comment. Table 2 has been revised to improve clarity and completeness.

Specifically, exact sample sizes have been provided for all studies where available, and population descriptions have been standardised to include key characteristics such as age and hearing status. In cases where information was limited, the most accurate available data have been reported.

These revisions enhance the transparency and allow a clearer assessment of the evidence.

• A few details are missing, like country and journal names. Study 22 (Laria et al.) is missing a country and journal name. Study 21 (Ross & Hoemann, 1975) is missing the journal
[Reply]: Thank you for your comment. We agree that complete reporting of study details is essential for clarity and transparency.

In response, Table 2 has been revised to include missing information on country and journal names where required. In particular, the entries for Laria et al. (2025) and Ross and Hoemann (1975) have been corrected to ensure that both country and journal details are consistently reported across all included studies.

These revisions improve the completeness and readability of the table.

• The results column is not consistent. Some are short, others are long. Make them all similar.
[Reply]: Thank you for your comment. We agree that consistency in the presentation of results is important for readability.

In response, the Results column in Table 2 has been revised to ensure a more consistent structure and level of detail across all studies. Descriptions have been standardised to provide concise and comparable summaries of the main findings.

These revisions improve the clarity and uniformity of the table.

• Kritzer (2009) is qualitative, but this was not listed in the methods. Fix this mismatch.
[Reply]: Thank you for your comment. We acknowledge that the inclusion of a qualitative study was not clearly specified in the original description of the eligibility criteria.

In response, the Methods section has been revised to explicitly state that both quantitative and qualitative observational studies were eligible for inclusion. We have also clarified the use of design-specific JBI critical appraisal tools according to study type.

These revisions ensure full consistency between the eligibility criteria and the included studies.

**Table 3 — Methodological Quality**
• Different checklists were used, but the same percentage rules were applied. This is not fully fair. Either adjust the percentages or describe quality in words (8-item JBI cross-sectional checklist vs 11-item case-control checklist vs Kritzer with the 10-item qualitative checklist)
[Reply]: Thank you for this important comment. We agree that applying identical percentage thresholds across different JBI checklists may not adequately account for differences in checklist structure and content. In response, we substantially revised the methodological quality assessment. Numerical thresholds and score-based categorizations were removed, and methodological quality is now interpreted through a qualitative framework based on individual JBI appraisal items and key methodological domains. In addition, Table 3 was completely redesigned to provide item-level reporting of all JBI assessments, including Yes/No/Unclear ratings and study-specific methodological considerations. This approach allows readers to directly evaluate the methodological strengths and limitations of each study without relying on percentage-based comparisons across different appraisal tools. These revisions improve transparency and align the quality assessment more closely with JBI guidance.

• One study -(Ross & Hoemann (1975)- was labeled experimental but assessed as cross-sectional. Explain this choice or fix it.
[Reply]: Thank you for this important comment. We agree that the original labeling of the study by Ross and Hoemann (1975) may have appeared inconsistent.

In the revised manuscript, this study has been consistently described as a comparative observational study (Table 2), as it does not involve experimental manipulation or random assignment but rather compares performance between deaf and hearing participants.

The use of the JBI Analytical Cross-Sectional Checklist was based on its methodological characteristics, in line with other comparative observational studies included in the review.

To further improve clarity and avoid potential ambiguity, we have carefully revised the manuscript to remove terminology that could imply an experimental design (e.g., “experimental tasks”) and replaced it with more appropriate expressions (e.g., “task-based assessments”) where relevant.

These revisions ensure full consistency between study design classification, methodological appraisal, and narrative description across all sections of the manuscript.

**Discussion**
• The authors did a good job in the discussion being the strongest part. The argument is clear and balanced.
[Reply]: Thank you for your positive and encouraging comment.

• Check the citation numbers carefully. Some do not match across sections.
[Reply]: Thank you for this important comment. We carefully reviewed all citation numbers throughout the manuscript and corrected inconsistencies to ensure full alignment across all sections, including the Abstract, Results, Tables, and Discussion.

• The main finding is repeated too many times. Say it clearly once, then support it.
[Reply]: Thank you for your comment. We agree that the main finding was reiterated across multiple sections of the Discussion.

In the revised manuscript, we have streamlined the Discussion to present the central finding clearly at the beginning, followed by a more structured synthesis of supporting evidence. Repetitive statements have been reduced or reformulated to avoid redundancy and improve the flow of the argument.

These revisions enhance the clarity, coherence, and overall readability of the Discussion.

• Dyscalculia is discussed early but not here. Either connect it or reduce it in the introduction.
[Reply]: Thank you for your comment. We agree that the role of developmental dyscalculia needed clearer integration within the Discussion.

In the revised manuscript, we have explicitly connected this concept to our findings by highlighting how the mathematical profile observed in DHH learners differs from that typically described in developmental dyscalculia, particularly in terms of the relative contribution of language-mediated versus core numerical processes.

In addition, we have streamlined the Introduction to clarify the role of developmental dyscalculia as a conceptual comparator, improving coherence between sections.

**Conclusion & Limitations**
• The conclusion is balanced and realistic. It ends with a useful message.
[Reply]: Thank you for your positive comment.

• Add two missing limitations. First, no grey literature or education databases. Second, the search filters may have been too strict.
[Reply]: Thank you for this important comment. We agree that these aspects were not sufficiently explicit in the previous version. In the revised manuscript, we have clarified that the search strategy did not include grey literature or education-specific databases, which may have limited the identification of some potentially relevant studies. In addition, we acknowledged that the use of strict inclusion criteria and search filters may have contributed to the exclusion of certain studies. These additions improve the transparency and completeness of the Limitations section.

• The final sentence is too strong. “Comprehensive” is not accurate for nine mixed studies. Use a more modest ending.

[Reply]: Thank you for this important comment. We agree that the final sentence in the previous version was too strong. In the revised manuscript, we have moderated the wording of the concluding statement to better reflect the scope, heterogeneity, and methodological limitations of the available evidence. This revision improves the accuracy and balance of the conclusion.

___________________________________________________________________________

**Reviewer #4:**

**General Comments**

The manuscript addresses an important topic and generally follows an appropriate systematic review methodology. However, I have several concerns regarding its technical robustness. First, there are inconsistencies in reporting the number of included studies throughout the manuscript, which raises concerns about the transparency of the study selection process. Second, the methodological quality assessment using the JBI critical appraisal tools requires further justification, particularly regarding the conversion of checklist outcomes into overall quality categories and risk-of-bias classifications. Third, substantial heterogeneity exists across the included studies in terms of participant characteristics, mathematical outcomes, and assessment methods, limiting the strength of the conclusions that can be drawn. Finally, some conclusions appear stronger than warranted by the available evidence, especially given the small number of included studies and the methodological limitations identified in several of them. Therefore, while the review is potentially valuable, major revisions are needed before I can conclude that the data fully support the authors’ conclusions.

**Major Comments**

1. Although the authors report the databases searched and provide general search strings, the search methodology lacks sufficient detail for full reproducibility. Specifically:

- The exact search dates should be reported consistently throughout the manuscript.

[Reply]: Thank you for this comment. We have carefully revised the manuscript to ensure consistent reporting of the search dates across all relevant sections.

The search period is now clearly and uniformly reported in both the Methods (Search Strategy section) and the Abstract, specifying that the literature search was conducted between March 31 and April 3, 2026, and included all studies published up to the date of the search.

- It is unclear whether database-specific adaptations of the search strategy were implemented.

[Reply]:	Thank you for this comment. We agree that clarification on this point is important for reproducibility.

In the revised manuscript, we have explicitly clarified that the search strategy was adapted to the specific characteristics of each database. In particular, controlled vocabulary (e.g., MeSH terms) was used where available (e.g., PubMed), while equivalent free-text terms and syntax were applied for other databases (Scopus and Web of Science), taking into account their indexing systems and search functionalities.

This clarification has been added to the Methods (Search Strategy section**)** to improve transparency and reproducibility.

- No information is provided regarding the use of filters, language restrictions, document-type restrictions, or manual reference screening.

[Reply]: Thank you for this important comment. We agree that detailed reporting of search restrictions is essential for reproducibility and transparency.

In the revised manuscript, we have clarified the use of filters and restrictions in the Methods (Search Strategy section). Specifically, we now explicitly report that only studies published in English were considered, and that filters related to observational study designs and publication types were applied where supported by the database interfaces, in accordance with the predefined eligibility criteria. Non-primary research articles (e.g., reviews, conference papers, and study protocols) were excluded during the initial screening phase.

We have also specified that the reference lists of included studies and relevant articles were systematically screened to identify additional eligible records.

These revisions improve the transparency and reproducibility of the search strategy.

- The authors should provide complete reproducible search strategies for each database, preferably as supplementary material.

[Reply]: Thank you for your suggestion. We agree that providing fully reproducible search strategies is essential for methodological transparency.

The complete search strings used for each database were already reported in detail in Table 1 in the original manuscript, allowing full reproducibility, including database-specific syntax and keywords.

To improve clarity, we have now explicitly stated in the Methods (Search Strategy section) that Table 1 contains the full search strategies applied for each database.

These clarifications further enhance the transparency and reproducibility of the review.

Because systematic reviews rely heavily on reproducible search procedures, additional detail is necessary.

2. There are several inconsistencies throughout the manuscript regarding the number and classification of included studies. For example:

- The Results section reports that eight studies met the eligibility criteria, whereas later sections repeatedly state that nine studies were included.

[Reply]: Thank you for this comment. We acknowledge that earlier versions of the manuscript contained inconsistencies in reporting the number of included studies.

We have carefully rechecked the study selection process and corrected all numerical discrepancies throughout the manuscript. The final number of included studies is twelve (n = 12), and this has now been consistently updated across all sections, including the Results, Abstract, PRISMA flow diagram, tables, and corresponding text.

These revisions ensure full internal consistency and improve the transparency of the study selection process.

- The PRISMA flow description should be carefully revised to ensure consistency across the text, figure, and tables.

[Reply]: Thank you for this comment. We acknowledge that inconsistencies were present in earlier versions of the PRISMA flow description.

In the revised manuscript, we have carefully rechecked and corrected the PRISMA flow diagram, the Results section, and all related tables to ensure full consistency across all stages of the study selection process. All numerical values, including the number of records identified, screened, excluded, and included, have been aligned throughout the text, figure, and tables.

These revisions improve the accuracy, transparency, and internal consistency of the study selection reporting.

- The numerical accounting of excluded studies at different screening stages should be verified because some totals do not appear to reconcile clearly.

[Reply]: Thank you for your comment. We have carefully rechecked the entire study selection process and revised the numerical reporting at each stage, including records identified, screened, excluded, and assessed for eligibility. All totals have been recalculated to ensure that they reconcile correctly across the PRISMA flow diagram, the Results section, and related tables. These revisions ensure full internal consistency and improve the transparency and accuracy of the study selection process.

These discrepancies raise concerns regarding the accuracy of the study selection process.

3. The rationale for excluding intervention studies, randomized trials, and clinical trials is not adequately explained. Since intervention studies may provide valuable information regarding mathematical performance in DHH populations, the authors should better justify their exclusion and explain how this decision aligns with the review objective. Similarly, the restriction to observational studies should be more explicitly justified.

[Reply]: Thank you for this important comment. We agree that intervention studies can provide valuable insights into mathematical performance in DHH populations. However, their exclusion in the present review was intentional and aligned with the specific objective of the study.

The aim of this systematic review was to examine the association between hearing status and mathematical performance in naturalistic contexts. For this reason, we focused exclusively on observational studies, which allow the investigation of mathematical abilities without the influence of experimental or intervention effects that may modify participants’ performance.

Intervention and clinical trial designs were therefore excluded because they assess outcomes under structured or modified conditions, which may introduce additional variables (e.g., training effects, intervention protocols) that limit comparability across studies and hinder the interpretation of baseline mathematical competencies.

To improve clarity, we have revised the Methods section to more explicitly justify the restriction to observational studies and to explain how this decision aligns with the research question and the intended scope of the review.

These revisions strengthen the methodological transparency and coherence of the study design.

4. The use of JBI critical appraisal tools is appropriate; however, the approach used to derive overall quality ratings is insufficiently described. Several concerns arise:

- The manuscript converts JBI checklist scores into categorical quality levels without providing references supporting this scoring procedure.

[Reply]: Thank you for this important comment. We agree that the conversion of JBI checklist outcomes into numerical scores or predefined categorical thresholds is not part of the Joanna Briggs Institute methodology. In response, we substantially revised the methodological quality assessment. Numerical cut-off values and score-based classifications were removed, and methodological quality is now interpreted through a qualitative framework based on individual JBI appraisal items and key methodological domains. In addition, Table 3 was completely redesigned to provide item-level reporting of all JBI assessments, including Yes/No/Unclear ratings and study-specific methodological notes supporting the appraisal process. This revision improves transparency, avoids the use of non-standard scoring procedures, and provides a more rigorous and reproducible assessment of methodological quality across the included studies.

- JBI tools were not originally designed to generate numerical quality scores.

[Reply]: Thank you for this comment. We agree that JBI critical appraisal tools were not designed to generate numerical quality scores. In response, we revised the methodological quality assessment to avoid score-based interpretations. Methodological quality is now evaluated using a qualitative framework based on individual JBI appraisal items and key methodological domains, rather than numerical scores or predefined thresholds. Furthermore, Table 3 was substantially redesigned to provide transparent item-level reporting of the appraisal process, including individual Yes/No/Unclear ratings and study-specific methodological considerations. These revisions improve transparency, align the appraisal process more closely with JBI guidance, and provide a more robust basis for interpreting methodological strengths and limitations across the included studies.

- The rationale for the selected cut-off values (≥70%, 50–69%, <50%) should be justified.

[Reply]: Thank you for this comment. We agree that the use of numerical cut-off values is not part of the recommended application of the JBI critical appraisal tools. In response, we removed all predefined thresholds and score-based categorizations from the methodological quality assessment. Rather than relying on percentage-based classifications, methodological quality is now interpreted through a qualitative assessment of individual JBI items and key methodological domains. Furthermore, Table 3 was substantially revised to provide transparent item-level reporting of the appraisal process, allowing readers to directly evaluate the methodological strengths and limitations of each included study. These revisions improve methodological transparency and align the quality assessment more closely with JBI guidance.

- The process used to determine overall risk-of-bias judgments remains unclear.

[Reply]: Thank you for this important comment. We agree that clarification of the process used to determine overall methodological appraisal judgments is essential.

In the revised manuscript, we clarified that these judgments were not derived from a fixed numerical scoring system. Instead, they were based on a qualitative assessment of individual JBI appraisal items and key methodological domains, including confounding control, validity of outcome measurement, and appropriateness of statistical analyses.

In addition, Table 3 was substantially redesigned to provide item-level reporting of all JBI assessments, including individual Yes/No/Unclear ratings and study-specific methodological considerations. This approach allows readers to directly evaluate the methodological strengths and limitations of each included study and provides a transparent basis for the methodological appraisal process.

These revisions improve the transparency, consistency, and interpretability of the quality assessment.

The authors should provide a stronger methodological justification or reconsider how quality assessments are summarized.

5. The Discussion repeatedly emphasizes methodological quality categories when interpreting study findings. However, no structured sensitivity analysis or evidence synthesis framework was used. The manuscript would benefit from a clearer explanation of how study quality influenced interpretation of the evidence and conclusions.

[Reply]: Thank you for this important comment. We agree that it is essential to clearly explain how methodological quality informed the interpretation of the findings. Because of the substantial heterogeneity across study designs, participant characteristics, and outcome measures, a formal sensitivity analysis was not feasible. Instead, the interpretation of findings was informed by the detailed methodological appraisal of each study. In the revised manuscript, the methodological quality assessment was substantially expanded and Table 3 was redesigned to report individual JBI appraisal items, their ratings (Yes/No/Unclear), and study-specific methodological considerations. This allowed methodological strengths and limitations to be explicitly considered during the narrative synthesis, with findings from studies presenting greater methodological limitations interpreted more cautiously. In addition, the manuscript now provides a clearer explanation of how methodological quality was incorporated into the overall interpretation of the evidence.

6. Although the authors evaluate methodological quality using JBI tools, no assessment of the overall certainty or strength of evidence is provided. While not mandatory, the inclusion of a framework such as GRADE or a justified alternative approach would substantially strengthen the interpretation of the findings and improve transparency regarding confidence in the conclusions.

[Reply]: Thank you for this insightful comment. We agree that frameworks such as GRADE can be valuable for assessing the overall certainty of evidence. However, after careful consideration, we concluded that a formal GRADE assessment was not appropriate for the present review due to the substantial heterogeneity across the included studies. Specifically, the studies differed considerably in design, participant characteristics, assessment instruments, mathematical domains evaluated, and outcome reporting. In addition, the limited number of studies available within individual mathematical domains and the absence of comparable quantitative outcome measures precluded a meaningful assessment of certainty across outcomes.

To address this issue, we revised the manuscript and explicitly added a justification for the non-use of GRADE in the Methodological Quality Assessment section. We also clarified that the overall strength of the evidence was evaluated through narrative synthesis, taking into account methodological quality, consistency of findings across studies, and key methodological limitations identified during critical appraisal. These revisions improve transparency regarding how confidence in the evidence was considered and interpreted.

7. The review includes only nine heterogeneous studies, several with small sample sizes and moderate methodological limitations. Therefore, statements suggesting that mathematical difficulties in DHH learners are primarily explained by linguistic accessibility should be presented more cautiously. The conclusions should better acknowledge the limited evidence base and avoid implying causal relationships that cannot be established from the included observational studies.

[Reply]: Thank you for this important comment. We agree that the interpretation of the findings should be carefully aligned with the strength and limitations of the available evidence.

In the revised manuscript, we have moderated the wording throughout the Discussion and Conclusion sections to avoid overinterpretation. In particular, statements regarding the role of linguistic accessibility in mathematical performance have been reframed using associative, rather than causal, language.

We have also strengthened the acknowledgment of the limited evidence base, explicitly highlighting the small number of included studies, their heterogeneity, and the presence of methodological limitations. In addition, the Conclusions section has been revised to emphasize that the findings should be interpreted with caution and considered indicative rather than definitive.

These revisions improve the overall balance, accuracy, and methodological appropriateness of the conclusions.

**Minor Comments**

1. The manuscript title uses the term "Deaf Patients", whereas most included studies concern children and students in educational contexts rather than clinical patients. The authors should consider replacing the term "patients" with a more appropriate population descriptor such as: "Mathematical Skills in Deaf and Hard-of-Hearing Children and Adolescents: A Systematic Review".

[Reply]: Thank you for this comment. We agree that the term “patients” is not appropriate for the population considered in this review.

In the revised manuscript, we have replaced the term “patients” with “deaf and hard-of-hearing children” in the title to better reflect the characteristics and context of the included studies. This change has also been applied consistently throughout the manuscript where relevant, to avoid unintended medicalization of the population.

2. The manuscript is generally understandable; however, several sections would benefit from careful English-language editing. Examples include: grammatical inconsistencies, punctuation issues, occasional typographical errors, repeated wording. A professional language revision is recommended.

[Reply]: Thank you for this comment. The English language has been carefully revised and improved throughout the manuscript by a native speaker.

3. The authors state that PRISMA 2020 guidelines were followed. However, several PRISMA items require further clarification, including: protocol registration details, screening procedures, handling of disagreements, assessment of reporting bias, justification for synthesis methods. The authors should ensure complete compliance with the PRISMA 2020 checklist.

[Reply]: Thank you for this important comment. We agree that full compliance with the PRISMA 2020 guidelines is essential to ensure transparency and methodological rigor.

In the revised manuscript, we have carefully reviewed and aligned the reporting with the PRISMA 2020 checklist. Specifically, we have clarified key methodological aspects, including protocol registration details, screening procedures, procedures for resolving disagreements, and the rationale for the selected synthesis approach.

We have also ensured consistency and completeness of reporting across all sections to fully meet PRISMA 2020 requirements.

These revisions enhance the transparency, reproducibility, and overall methodological rigor of the manuscript.

4. Tables 2 and 3 are informative but would benefit from improved formatting and standardization. Several study descriptions are overly detailed, whereas others provide insufficient information regarding sample characteristics and outcome measures.

[Reply]: Thank you for this helpful comment. We agree that improving the consistency and clarity of Tables 2 and 3 enhances the overall readability of the manuscript.

In the revised version, we have standardized the formatting of both tables and revised the study descriptions to ensure a more balanced and consistent level of detail across all entries. In particular, overly detailed descriptions have been streamlined, while missing information on sample characteristics and outcome measures has been added where available.

These revisions improve the clarity, comparability, and overall readability of the tables.

**Additional comments in main text**

To ensure a comprehensive and rigorous evaluation of the manuscript, the complete peer-review comments, including specific technical assessments and detailed editorial recommendations, have been systematically compiled and uploaded as a separate attached document. Please consult the attached file for the full report.

**Comment 1:** The title *"Mathematical Skills in Deaf Patients: A Systematic Review"* and the repeated use of the word *"patients"* throughout the manuscript are highly problematic. The 9 studies included in your review assess children and adolescents in naturalistic, educational, or home-based environments, focusing on cognitive development and academic math learning. They are not medical patients undergoing clinical trials or medical treatments for their mathematical skills. Referring to DHH children as "patients" pathologizes a pedagogical and cognitive issue.

**[Reply]:** We thank the Reviewer for this thoughtful and important comment.

We fully agree that the use of the term “patients” is inappropriate in the context of this manuscript.

The studies included in our review examine deaf and hard-of-hearing (DHH) children and adolescents in educational, cognitive, and family contexts rather than clinical settings, and the use of medicalized terminology does not accurately reflect the nature of the populations or the research paradigms involved.

We also appreciate the Reviewer’s observation that such terminology may inadvertently pathologize what are fundamentally pedagogical and developmental issues. In line with this perspective, we have revised the manuscript throughout, replacing “patients” with more appropriate terms such as “children,” and hard-of-hearing (DHH) learners,” depending on context.

The title has also been modified accordingly.

We believe these changes improve both the conceptual clarity and the alignment of the manuscript with current perspectives in Deaf Education and developmental research.

**Comment 2:** you state: *"The study protocol has been stored at the following link: '*[*https://archive.org/details/osf-registrations-fbk98-v1*](https://www.google.com/search?q=https://archive.org/details/osf-registrations-fbk98-v1&authuser=2)*, with registration doi:* [*https://doi.org/10.17605/OSF.IO/EQXMZ*](https://doi.org/10.17605/OSF.IO/EQXMZ)*."*

While the OSF DOI (EQXMZ) correctly points to the registration of this math systematic review, the accompanying Internet Archive link is an obvious copy-paste error from your team's previous work. The registration ID fbk98 (and its corresponding DOI 10.17605/OSF.IO/FBK98) belongs to a completely different systematic review on adult bimodal acoustic stimulation published by several of the same authors in 2024.

- Required Action: You must correct this editorial error. Please update the Internet Archive link to the correct archive file matching the current review on mathematical skills, or remove the incorrect archive link to avoid misleading readers.

**[Reply]:** Thank you for this important comment. We acknowledge that the Internet Archive link in the previous version was an editorial error resulting from a copy-paste mistake.

This issue has already been corrected in the revised manuscript, where the incorrect link has been removed and the protocol is now accurately reported using the appropriate OSF registration DOI.

This correction ensures clarity and prevents potential confusion for readers.

**Comment 3:** Fatal Contradiction Regarding JBI Tool Applied to Laria C. et al. (2025): There is a severe mismatch between your Methods narrative and Table 3. On Page 12 of the manuscript, you explicitly state: *"The JBI Critical Appraisal Checklist for Analytical Cross-Sectional Studies was applied to eight quantitative observational studies..."* and you explicitly include *Laria et al. (2025)* in this list. However, in Table 3, *Laria C. et al. (2025)* is suddenly classified as a "Case-control study" and appraised using the "Case-control checklist". This is a major internal contradiction that must be resolved.

**Required action:** Resolve the design of Laria et al. (2025): Remove *Laria et al. (2025)* from the list of cross-sectional studies to ensure consistency.

**[Reply]:** Thank you for this important comment. We acknowledge that this inconsistency was present in an earlier version of the manuscript.

This issue has already been addressed in the revised version. We confirm that Laria et al. (2025) is correctly classified as a case–control study, and we have ensured full consistency across all sections of the manuscript. Specifically, the Methods section has been corrected to remove this study from the list of cross-sectional studies, and the study design is now consistently reported as case–control in the text and tables.

These revisions resolve the discrepancy and ensure full internal consistency.

**Comment 4:** There is a mathematical mismatch in the narrative description of the preliminary exclusion phase on Page 13. You write:

*"In the first step we removed 40 articles. In particular, 22 articles were excluded due to the study design... 18 were in other languages..."*

*"Following preliminary exclusions, 537 records underwent title and abstract screening."*

While 22 + 18 = 40 matches your described exclusions, the mathematics from the initial database search total does not add up:

Please pay attention to this consistency from the beginning to the end of the manuscript, including in figures or tables.

**[Reply]:** Thank you for this comment. We acknowledge that inconsistencies were present in the numerical description of the preliminary exclusion phase in a previous version of the manuscript.

In the revised manuscript, we have carefully rechecked the numerical reporting at each stage of the study selection process. All values have been corrected to ensure that they are mathematically consistent and fully aligned across the narrative description, the PRISMA flow diagram, and the corresponding tables.

These revisions ensure full internal consistency and improve the clarity of the study selection reporting.

**Comment 5:** *"As a result, eight studies met all eligibility criteria and were included in the final synthesis. As a result, our systematic search provided 9 articles."*

This is a direct and confusing contradiction within the same paragraph.

Required Action: Please resolve this discrepancy. Ensure that you consistently state that 9 studies were included throughout the entire manuscript, as Table 2, Table 3, and the PRISMA flowchart all report 9 studies.

**[Reply]:** Thank you for this comment. We acknowledge that this inconsistency was present in an earlier version of the manuscript.

In the revised manuscript, we have corrected all discrepancies related to the number of included studies. The final number of included studies is twelve (n = 12), and this has been reported consistently throughout the manuscript, including the Results section, tables, and PRISMA flow diagram.

These revisions ensure full internal consistency and eliminate any ambiguity in the reporting.

**Comment 6:** According to the PRISMA flowchart (Fig 1), you also removed *"Duplicate reports not retrieved (n = 2)"*. However, this is completely omitted from the main text narrative.

Required Action: Revise the text to explicitly account for the 2 duplicate reports. Clarify that a total of 42 articles were removed in the preliminary stage (40 via exclusion criteria and 2 duplicates) to make the step-by-step reduction to 537 mathematically transparent to

**[Reply]:** Thank you for this comment. We acknowledge that duplicate records were not explicitly reported in the narrative description in the previous version of the manuscript.

In the revised manuscript, we have clarified this point by explicitly reporting the removal of duplicate records (n = 2) alongside other preliminary exclusions (n = 40), ensuring consistency with the PRISMA flow diagram.

**Comment 7**: Incomplete Data Extraction (Table 2)

For several of the included studies in Table 2, you have failed to extract and report basic quantitative sample sizes (n), using vague qualitative phrases instead:

- Titus (1995): *"Moderate sample... exact number varies by subgroup"*
- Zarfaty et al. (2004): *"Small sample (exact not consistently reported in summaries...)"*
- Lang et al. (2007): *"exact n not given in abstract"*

Stating that the exact sample size was "not consistently reported in summaries" or "not given in abstract" strongly suggests that you only reviewed the abstracts/summaries of these papers rather than retrieving and reading the peer-reviewed full-texts. In a rigorous systematic review, extracting basic sample sizes from the full-text is a mandatory requirement.

Required Action: Retrieve the full-text PDF files for Titus (1995), Zarfaty et al. (2004), and Lang et al. (2007). Extract the exact sample sizes (for both DHH and control groups) and replace the vague text in Table 2 with precise numerical values

**[Reply]:** Thank you for this important comment. We acknowledge that the previous version of Table 2 included incomplete information regarding sample sizes for some studies.

In the revised manuscript, we have carefully reviewed the full-text articles and updated Table 2 to report precise sample sizes (n) for all included studies, including Titus (1995), Zarfaty et al. (2004), and Lang et al. (2007).

These revisions ensure completeness and accuracy of data extraction and improve the transparency of the review.

**Comment 8:** For transparency and in alignment with PLOS ONE's strict ethics and disclosure policies, you must explicitly declare this intellectual/non-financial conflict of interest in the *Conflicts of Interest* section. Additionally, please describe the specific measures taken to ensure objectivity during the screening, data extraction, and critical appraisal of your own study (e.g., whether the critical appraisal of Laria et al. was conducted independently by co-authors who were not involved in that primary study).

**[Reply]:** Thank you for this important comment. We agree that full transparency regarding potential conflicts of interest is essential.

In the revised manuscript, we have explicitly declared the presence of a non-financial (intellectual) conflict of interest in the Conflicts of Interest section, as one of the included studies (Laria et al., 2025) was authored by members of the review team.

To ensure objectivity and minimize potential bias, we have also added a clarification in the Methods section (Study Selection and Data Extraction) specifying that the screening, data extraction, and methodological quality assessment of this study were conducted by reviewers who were not involved in the original study.

These revisions improve transparency and ensure alignment with ethical and reporting standards.

**Comment 9:** Invalid Checklist Applied to Ross B. M. (1975): *Ross B. M. (1975)* is described as a "comparative experimental" study but was appraised using the *JBI Analytical Cross-Sectional Checklist* (scoring 3/8). Applying an observational cross-sectional tool to an experimental design is methodologically invalid because cross-sectional checklists do not evaluate experimental risk of bias (such as pre-post intervention measurements, group allocation, or intervention administration).

Required Action: Re-appraise Ross B. M. (1975): Re-evaluate this study using the appropriate *JBI Quasi-Experimental Critical Appraisal Checklist* (which contains 9 items). Update Table 3 and the Results narrative with the new score and denominator.

**[Reply]:** Thank you for this important comment. We acknowledge that the terminology used in an earlier version of the manuscript may have suggested an experimental design.

This issue has already been addressed in the revised manuscript. Ross and Hoemann (1975) is now consistently described as a comparative observational study across all sections, and terminology suggesting an experimental design has been removed.

Accordingly, the use of the JBI Analytical Cross-Sectional Checklist is appropriate for this study, as it does not involve any intervention, group allocation, or experimental manipulation, but rather compares naturally occurring groups.

These revisions ensure methodological consistency and avoid any ambiguity in study classification.

**Comment 10:** Complete Absence of Case-Control Checklist Description in the Text: While you detailed the appraisal domains for the Analytical Cross-Sectional Checklist (Page 7) and the Qualitative Checklist (Page 8), there is absolutely no narrative description, list of criteria, or mention of the JBI Case-Control Checklist in your Methods text, despite it appearing prominently in Table 3.

Req: Update the Methods narrative: Officially add the JBI Case-Control Checklist description and its 10 specific items into your Methods text on Page 8.

**[Reply**]: Thank you for this important comment. We acknowledge that the description of the JBI Case-Control Checklist was not included in the Methods section in an earlier version of the manuscript.

In the revised manuscript, we have addressed this issue by adding a detailed description of the JBI Case-Control Checklist, including its key appraisal domains, in the Methods section (JBI Critical Appraisal Tools subsection).

This addition ensures consistency with the other JBI tools described and improves the completeness and transparency of the methodological reporting.

**Comment 11**: Incorrect Denominator for the Case-Control Checklist (Table 3): In Table 3, *Laria C. et al. (2025)* is reported to have met 8/11 criteria. This is mathematically and methodologically impossible because the official *JBI Critical Appraisal Checklist for Case-Control Studies* consists of exactly 10 questions/items. The 11-item checklist belongs to Cohort Studies. This indicates you either used the wrong checklist while mislabeling it, or reported an incorrect denominator.

Required action: Re-appraise Laria et al. (2025): Re-evaluate the study using the correct 10-item JBI Case-Control Checklist, and update Table 3 with the accurate score and denominator (out of 10, not 11).

**[Reply]:** Thank you for this important comment. We acknowledge that the denominator reported for Laria et al. (2025) in an earlier version of Table 3 was incorrect. In the revised manuscript, we corrected the application of the JBI Case-Control Checklist and revised the methodological appraisal accordingly. Table 3 was substantially redesigned and now reports the individual checklist items, their appraisal (Yes/No/Unclear), and study-specific methodological considerations, ensuring methodological accuracy, consistency, and transparency in the quality assessment process. These revisions improve the clarity of the appraisal and ensure consistency in the application of the JBI critical appraisal tools across all included studies.

**Comment 12:** This systematic review includes and appraises a primary case-control study by Laria C. et al. (2025) published in *Hearing Balance and Communication*. The authors of that primary study (Carla Laria, Nicola Serra, Rita Malesci, Anna Rita Fetoni) are also the core authors of this systematic review. However, on this Page you declare:

*"The authors declare no conflicts of interest."*

Evaluating your own primary research within your own systematic review is a classic non-financial conflict of interest (intellectual conflict of interest) that can introduce researcher allegiance bias.

Required Action: For transparency and in alignment with PLOS ONE's strict ethics and disclosure policies, you must explicitly declare this intellectual/non-financial conflict of interest in the *Conflicts of Interest* section. Additionally, please describe the specific measures taken to ensure objectivity during the screening, data extraction, and critical appraisal of your own study (e.g., whether the critical appraisal of Laria et al. was conducted independently by co-authors who were not involved in that primary study).

**[Reply]:** Thank you for this important comment. We agree that full transparency regarding potential conflicts of interest is essential.

As addressed in response to the related comment, we have revised the manuscript to explicitly declare the presence of a non-financial (intellectual) conflict of interest in the Conflicts of Interest section, as one of the included studies (Laria et al., 2025) was authored by members of the review team.

We have also clarified in the Methods section (Study Selection and Data Extraction) the procedures adopted to ensure objectivity, specifying how the evaluation of this study was handled to minimize potential bias.

These revisions improve transparency and ensure alignment with ethical and reporting standards.
