# Supplementary material for: Mathematical skills in deaf and hard-of-hearing children: A systematic review
Source: PLoS One. 2026 Sep 16;21(9):e0358324. doi: 10.1371/journal.pone.0358324 (PMC13581004; doi:10.1371/journal.pone.0358324)
Supplement: Peer review history

# Peer review: Decision Letter 0

**DOI:** 10.1371/journal.pone.0358324.r001

**Authors:** Rana Alkhamra

10 Jun 2026

Dear Dr. Serra,

plosone@plos.org. When you're ready to submit your revision, log on to https://www.editorialmanager.com/pone/ and select the 'Submissions Needing Revision' folder to locate your manuscript file.

- A letter that responds to each point raised by the academic editor and reviewer(s). You should upload this letter as a separate file labeled 'Response to Reviewers'.
- A marked-up copy of your manuscript that highlights changes made to the original version. You should upload this as a separate file labeled 'Revised Manuscript with Track Changes'.
- An unmarked version of your revised paper without tracked changes. You should upload this as a separate file labeled 'Manuscript'.

If you would like to make changes to your financial disclosure, please include your updated statement in your cover letter. Guidelines for resubmitting your figure files are available below the reviewer comments at the end of this letter.

If applicable, we recommend that you deposit your laboratory protocols in protocols.io to enhance the reproducibility of your results. Protocols.io assigns your protocol its own identifier (DOI) so that it can be cited independently in the future. For instructions see: https://journals.plos.org/plosone/s/submission-guidelines#loc-laboratory-protocols. Additionally, PLOS ONE offers an option for publishing peer-reviewed Lab Protocol articles, which describe protocols hosted on protocols.io. Read more information on sharing protocols at https://plos.org/protocols?utm_medium=editorial-email&utm_source=authorletters&utm_campaign=protocols.

As the corresponding author, your ORCID iD is verified in the submission system and will appear in the published article. PLOS supports the use of ORCID, and we encourage all coauthors to register for an ORCID iD and use it as well. Please encourage your coauthors to verify their ORCID iD within the submission system before final acceptance, as unverified ORCID iDs will not appear in the published article. *Only*  the individual author can complete the verification step; PLOS staff *cannot*  verify ORCID iDs on behalf of authors.

We look forward to receiving your revised manuscript.

Kind regards,

Rana Alkhamra

Academic Editor

PLOS One

Journal Requirements:

When submitting your revision, we need you to address these additional requirements.

1. Please ensure that your manuscript meets PLOS ONE's style requirements, including those for file naming. The PLOS ONE style templates can be found at

https://journals.plos.org/plosone/s/file?id=wjVg/PLOSOne_formatting_sample_main_body.pdf and

https://journals.plos.org/plosone/s/file?id=ba62/PLOSOne_formatting_sample_title_authors_affiliations.pdf

2. We note that your Data Availability Statement is currently as follows: “All data underlying the findings of this study are included within the manuscript and its Supporting Information files. No new datasets were generated during the current systematic review.”

Please confirm at this time whether or not your submission contains all raw data required to replicate the results of your study. Authors must share the “minimal data set” for their submission. PLOS defines the minimal data set to consist of the data required to replicate all study findings reported in the article, as well as related metadata and methods (https://journals.plos.org/plosone/s/data-availability#loc-minimal-data-set-definition).

For example, authors should submit the following data:

- The values behind the means, standard deviations and other measures reported;

- The values used to build graphs;

- The points extracted from images for analysis.

Authors do not need to submit their entire data set if only a portion of the data was used in the reported study.

If your submission does not contain these data, please either upload them as Supporting Information files or deposit them to a stable, public repository and provide us with the relevant URLs, DOIs, or accession numbers. For a list of recommended repositories, please see https://journals.plos.org/plosone/s/recommended-repositories.

If there are ethical or legal restrictions on sharing a de-identified data set, please explain them in detail (e.g., data contain potentially sensitive information, data are owned by a third-party organization, etc.) and who has imposed them (e.g., an ethics committee). Please also provide contact information for a data access committee, ethics committee, or other institutional body to which data requests may be sent. If data are owned by a third party, please indicate how others may request data access.

3.  Please include captions for your Supporting Information files at the end of your manuscript, and update any in-text citations to match accordingly. Please see our Supporting Information guidelines for more information: http://journals.plos.org/plosone/s/supporting-information.

4. If the reviewer comments include a recommendation to cite specific previously published works, please review and evaluate these publications to determine whether they are relevant and should be cited. There is no requirement to cite these works unless the editor has indicated otherwise.

[Note: HTML markup is below. Please do not edit.]

Reviewers' comments:

Reviewer's Responses to Questions

**Comments to the Author**

1. Is the manuscript technically sound, and do the data support the conclusions?

Reviewer #1: Yes

Reviewer #2: Partly

Reviewer #3: Yes

Reviewer #4: Partly

**********

2. Has the statistical analysis been performed appropriately and rigorously?

Reviewer #1: Yes

Reviewer #2: N/A

Reviewer #3: N/A

Reviewer #4: No

**********

3. Have the authors made all data underlying the findings in their manuscript fully available?

The PLOS Data policy

Reviewer #1: Yes

Reviewer #2: Yes

Reviewer #3: Yes

Reviewer #4: No

**********

4. Is the manuscript presented in an intelligible fashion and written in standard English?

Reviewer #1: Yes

Reviewer #2: Yes

Reviewer #3: Yes

Reviewer #4: No

**********

Reviewer #1: Title: Mathematical Skills in Deaf Patients: A Systematic Review

Journal: ??

Manuscript ID: PONE-D-26-21292

Summary:

This manuscript is a literature review of studies conducted to examine the mathematics performance of Deaf children under the age of 18 years of age. Using a highly restrictive set of review criteria, the body of mathematics research in the field was reduced to a set of 9 studies. A review of these studies indicates that hearing loss itself is not a cause of low mathematics performance, but rather the low language and educational situations that may accompany the hearing loss.

Strengths:

Mathematics is an under-researched area of study in Deaf Education so research that focuses on this area is much needed. The manuscript is interesting and well written.

Areas of Concern:

• The reference to the Deaf individuals under focus in this manuscript as “patients” in the title is concerning to this reviewer. In every study reviewed in this manuscript, the participants were children. The children were students at a school, or members of a family- they were not sick, and they were not in a hospital. Please find a new word.

• By using such highly restrictive criteria, many good studies were eliminated from review that might have provided good information.

• The authors state in the conclusion that “These limitations highlight the need for more robust and methodologically rigorous research, including clearer descriptions of linguistic and cognitive profiles and standardized assessments targeting distinct mathematical domains.” While this reviewer does not doubt the validity of this statement, it is important to remember the characteristics of the population that one is working with. Deaf children are a very heterogenous population. Cognitive profiles are dependent on language, and these are often difficult to determine when children are young. Then, in using standardized assessments, whom should these assessments be standardized on? Hearing children? Tests standardized for hearing children are often not appropriate for deaf children, and tests created for Deaf children are difficult to develop because of the heterogeneity of the population. The challenges to creating rigorous research are numerous in Deaf Education. Nevertheless, there is at least one study out there, that this reviewer knows of, that did use a standardized assessment- for a population in the appropriate age range that was not included in the literature review for this manuscript. Not sure why.

Reviewer #2: The topic is highly relevant to the fields of mathematics education, special education, and cognitive development. Overall, the study addresses an important gap in synthesizing evidence on domain-specific mathematical abilities and the role of linguistic access in DHH learners.

Major strengths

The review demonstrates several strengths. First, it follows PRISMA 2020 guidelines and uses a predefined protocol registered on OSF, which enhances transparency and reproducibility. Second, the search strategy is comprehensive, covering major databases (PubMed, Scopus, Web of Science) and employing both controlled vocabulary and free-text terms. Third, the inclusion of a structured quality appraisal using JBI tools adds methodological rigor and allows for a nuanced interpretation of study quality. Finally, the narrative synthesis is well-developed and appropriately highlights the heterogeneity of findings, particularly the distinction between linguistic and non-linguistic mathematical tasks.

Major concerns

Despite these strengths, several methodological and reporting issues require attention:

Inconsistencies in study selection reporting

There is inconsistency in the number of included studies. The manuscript reports both eight and nine included studies in different sections. Additionally, the PRISMA flow reporting contains numerical inconsistencies (e.g., 579 records minus exclusions does not consistently lead to the reported final sample). These discrepancies should be carefully verified and corrected.

Clarification of eligibility criteria

The inclusion/exclusion criteria are detailed but somewhat repetitive and occasionally unclear (e.g., overlapping descriptions of study designs). A more concise and structured presentation of PICOS criteria would improve clarity.

Lack of quantitative synthesis justification

Although a meta-analysis is not required, the authors should more explicitly justify the absence of quantitative synthesis. Given that some included studies report comparable outcomes, a brief discussion of effect size heterogeneity or feasibility of meta-analysis would strengthen methodological transparency.

Quality appraisal interpretation

While JBI tools are appropriately applied, the categorization of risk of bias (low/moderate/high) appears partially subjective and not fully standardized. A clearer explanation of thresholds and consistency across different JBI checklists is needed. Additionally, combining qualitative and quantitative studies under similar appraisal logic may require further justification.

Heterogeneity of included studies

The included studies vary substantially in design, sample size, age range, and outcome measures. Although this is acknowledged in the discussion, a more structured subgroup interpretation (e.g., by age group, task type, or communication modality) would improve analytical depth.

Older studies and methodological limitations

Several included studies are dated (e.g., 1975), and their methodological limitations are acknowledged. However, their influence on overall conclusions should be more critically weighted, as older methodologies may not be fully comparable to contemporary research standards.

Language and reporting clarity

Minor grammatical inconsistencies and repetition appear throughout the manuscript (e.g., repeated phrases in Methods and Results sections). A careful language revision is recommended to improve readability and precision.

Minor comments

The PRISMA flow description should be aligned with the final included study count.

Table formatting (especially Table 2) would benefit from standardization for readability.

Some methodological terms (e.g., “moderate–high quality”) require clearer operational definitions.

Keywords could be refined to better align with indexing databases.

Ethical and publication considerations

No major ethical concerns are identified, as the study is based on published literature. However, the authors should ensure full transparency regarding protocol adherence and clarify whether any deviations from the registered protocol occurred. Additionally, consistency in reporting is essential to avoid concerns regarding data handling or selective inclusion.

Conclusion

This systematic review addresses an important and underexplored area with clear relevance to education and developmental psychology. With revisions to improve consistency, methodological transparency, and reporting clarity, the manuscript has the potential to make a meaningful contribution to the field.

Reviewer #3: Introduction

The manuscript entitled “Mathematical Skills in Deaf Patients: A Systematic Review” offers a valuable and timely contribution to the growing body of literature examining cognitive and educational outcomes among deaf individuals. By synthesizing existing research on mathematical performance within this population, the authors provide important insights into both the challenges and potential strengths associated with numerical cognition in deaf learners. The review is particularly commendable for its thorough methodology and its well-developed discussion section, which thoughtfully interprets findings and situates them within broader educational and cognitive frameworks.

Overall, the authors have demonstrated considerable effort in compiling and analyzing relevant studies, and their work has clear implications for both research and practice. However, despite these strengths, there remain several issues that should be addressed to further improve the clarity, rigor, and impact of the manuscript. These concerns are outlined below.

Title & Abstract

• The title is precise and reflect the content, nevertheless, using the term “Deaf Patients” does not match the focus. The review is about children in education. It would fit better to say “Deaf and hard-of-hearing children and adolescents” or “DHH learners.”

• The abstract is clear, but it is missing key details; Mainly, how many databases you searched, the time period for the study, and the reason for the use of narrative synthesis instead of a meta-analysis.

• According to the criteria, the paper included only nine studies, and their quality varies. I think the researchers should be careful in presenting the results where it sounded stronger than they are.

Introduction

• The reason for the study is clear. The link between math skills and daily life is easy to understand.

• The section on developmental dyscalculia gets quite a lot of space relative to its role in the review. Further explanation is needed clearly why it's included as a comparator — and why wasn’t this issue featured in the discussion.

• The introduction would benefit from a sharper statement of what gap in the existing systematic evidence this review is actually filling. It's implied, but never said directly.

Methods

• The protocol statement needs fixing. The link is broken and the format is unclear. Include the platform, number, date, and whether it was registered before screening. Remove the extra “ù” symbol before the Inclusion Criteria and Eligibility.

• The research question — "What is the impact of deafness on the development of mathematical skills?" — is too broad and implies CAUSALITY that observational studies can't establish. Since your criteria limit the population to under 18 years, the question should ask about the association between DHH and mathematical skill development among DHH children and adolescents?" this might be more appropriate and safer about the study designs involved.

• The eligibility criteria need a few clarifications:

o Were both congenital and acquired hearing loss included?

o Was the severity of hearing loss a factor?

o The phrase "when applicable" for the comparator group is vague — were studies without a comparison group eligible or not?

o The outcome criterion is broad; the researchers needed to be specific about which cognitive processes counted and how they relate to mathematical outcomes.

• The search strategy is well reported. But using terms like “cohort” or “cross-sectional” may have excluded studies. Either explain this choice or remove those filters Adding ERIC, PsycINFO, EBSCO, or Education Source would also strengthen the coverage for this field.

• The study selection process is described well, but the data extraction process is barely mentioned. Readers need to know who extracted the data, whether it was done independently or checked by a second person, what form was used, and how disagreements were resolved. If inter-rater reliability was calculated, report it.

• The quality appraisal is mostly appropriate, nevertheless, it was mentioned that cross-sectional checklist has nine items but list eight, this inconsistency needs to be fixed. The phrase "moderate expertise" for the reviewers slightly undermines confidence in the process, use more appropriate words focusing mainly on the training rather than the level. Furthermore, Also clarify how disagreements were resolved before involving the third reviewer.

• Your quality scoring is not standard for JBI. Either justify it or say you created it. It is better to say “higher,” “moderate,” or “lower quality.”

• One qualitative study was included, but this is not in the criteria. If qualitative studies were allowed, say so. If not, explain why it was included.

Results — Screening & Flow Diagram

• The numbers are inconsistent. You say eight studies in one place and nine in another. Use one number everywhere.

• The PRISMA diagram is useful. But do not mix duplicate removal with other exclusions. Also, “not retrieved” is confusing.

• "Does not match with our topic" as the reason for excluding 500 records is too vague. Even a brief list of the main reasons at that stage — wrong population, wrong outcome, wrong design — would make the screening process more transparent and reproducible.

Table 2 — Study Descriptions

• Several studies are listed without exact sample sizes. Try to include numbers for all. If estimated please mention it. Leaving it blank makes it very hard to assess the weight of evidence.

• A few details are missing, like country and journal names. Study 22 (Laria et al.) is missing a country and journal name. Study 21 (Ross & Hoemann, 1975) is missing the journal

• The results column is not consistent. Some are short, others are long. Make them all similar.

• Kritzer (2009) is qualitative, but this was not listed in the methods. Fix this mismatch.

Table 3 — Methodological Quality

• Different checklists were used, but the same percentage rules were applied. This is not fully fair. Either adjust the percentages or describe quality in words (8-item JBI cross-sectional checklist vs 11-item case-control checklist vs Kritzer with the 10-item qualitative checklist)

• One study -(Ross & Hoemann (1975)- was labeled experimental but assessed as cross-sectional. Explain this choice or fix it.

Discussion

• The authors did a good job in the discussion being the strongest part. The argument is clear and balanced.

• Check the citation numbers carefully. Some do not match across sections.

• The main finding is repeated too many times. Say it clearly once, then support it.

• Dyscalculia is discussed early but not here. Either connect it or reduce it in the introduction.

Conclusion & Limitations

• The conclusion is balanced and realistic. It ends with a useful message.

• Add two missing limitations. First, no grey literature or education databases. Second, the search filters may have been too strict.

• The final sentence is too strong. “Comprehensive” is not accurate for nine mixed studies. Use a more modest ending.

Reviewer #4: To ensure a comprehensive and rigorous evaluation of the manuscript, the complete peer-review comments, including specific technical assessments and detailed editorial recommendations, have been systematically compiled and uploaded as a separate attached document. Please consult the attached file for the full report.

**********

what does this mean?). If published, this will include your full peer review and any attached files.

If you choose “no”, your identity will remain anonymous but your review may still be made public.

**Do you want your identity to be public for this peer review?**  For information about this choice, including consent withdrawal, please see our Privacy Policy

Reviewer #1: No

Reviewer #2: No

Reviewer #3: **Yes:**  Hatem Alkhamra

Reviewer #4: **Yes:**  Wenda Alifulloh

**********

[NOTE: If reviewer comments were submitted as an attachment file, they will be attached to this email and accessible via the submission site. Please log into your account, locate the manuscript record, and check for the action link "View Attachments". If this link does not appear, there are no attachment files.]

To ensure your figures meet our technical requirements, please review our figure guidelines: https://journals.plos.org/plosone/s/figures

You may also use PLOS’s free figure tool, NAAS, to help you prepare publication quality figures: https://journals.plos.org/plosone/s/figures#loc-tools-for-figure-preparation.

NAAS will assess whether your figures meet our technical requirements by comparing each figure against our figure specifications.

Attachment Submitted filename: Peer Review Report.docx

Attachment Submitted filename: Comment - Systematic review -math skills-deaf_Final_ver3.docx

# Author comment: Author response to Decision Letter 1

**DOI:** 10.1371/journal.pone.0358324.r002

4 Aug 2026

Review Comments to the Author

Reviewer #1:

Summary:

This manuscript is a literature review of studies conducted to examine the mathematics performance of Deaf children under the age of 18 years of age. Using a highly restrictive set of review criteria, the body of mathematics research in the field was reduced to a set of 9 studies. A review of these studies indicates that hearing loss itself is not a cause of low mathematics performance, but rather the low language and educational situations that may accompany the hearing loss.

Strengths:

Mathematics is an under-researched area of study in Deaf Education so research that focuses on this area is much needed. The manuscript is interesting and well written.

[Reply]: We thank the Reviewer for these positive comments and for highlighting the relevance of the topic.

We agree that mathematics in deaf and hard-of-hearing (DHH) populations remains a relatively under-investigated area within Deaf Education, and one of the motivations of this review was to contribute to addressing this gap in the literature.

We also appreciate the Reviewer’s acknowledgment of the clarity and readability of the manuscript.

Areas of Concern:

• The reference to the Deaf individuals under focus in this manuscript as “patients” in the title is concerning to this reviewer. In every study reviewed in this manuscript, the participants were children. The children were students at a school, or members of a family- they were not sick, and they were not in a hospital. Please find a new word.

[Reply]: Thank you for your suggestion. Accordingly, we have revised the title to replace the term “patients” with “children”, in order to better reflect the characteristics and context of the populations included in the review. This change has also been applied throughout the manuscript where relevant, to ensure consistency and to avoid unintended medicalization of the study population.

• By using such highly restrictive criteria, many good studies were eliminated from review that might have provided good information.

[Reply]: Thank you for this insightful comment. We acknowledge that the use of restrictive inclusion criteria may have led to the exclusion of potentially relevant studies.

However, the inclusion criteria were intentionally defined a priori, in accordance with the PICOS framework and PRISMA 2020 guidelines, to ensure methodological consistency and comparability across studies. In particular, we limited the review to observational studies involving participants under 18 years of age and requiring direct assessment of mathematical skills. This approach was chosen to specifically investigate the relationship between hearing status and mathematical performance in naturalistic contexts, without the confounding influence of intervention effects or indirect outcome measures.

To address the reviewer’s concern and improve clarity, we have made the following revisions to the manuscript:

- We clarified the observational nature of the included studies in the Abstract (Methods section), explicitly stating that this is a systematic review of observational studies.

- We refined the study aim in the Introduction by specifying “observational methodological approaches,” ensuring full alignment between the stated objective and the inclusion criteria.

- We added a statement at the end of the Inclusion Criteria section to explicitly justify the rationale for adopting stricter selection criteria in terms of methodological consistency, internal validity, and interpretability of findings.

- We further acknowledged this aspect in the Limitations section, noting that strict inclusion criteria may have resulted in the exclusion of potentially relevant studies, while emphasizing that this decision was made to enhance methodological homogeneity.

While this approach may have reduced the number of eligible studies, it allowed for a more focused and coherent synthesis of the available evidence.

• The authors state in the conclusion that “These limitations highlight the need for more robust and methodologically rigorous research, including clearer descriptions of linguistic and cognitive profiles and standardized assessments targeting distinct mathematical domains.” While this reviewer does not doubt the validity of this statement, it is important to remember the characteristics of the population that one is working with. Deaf children are a very heterogenous population. Cognitive profiles are dependent on language, and these are often difficult to determine when children are young. Then, in using standardized assessments, whom should these assessments be standardized on? Hearing children? Tests standardized for hearing children are often not appropriate for deaf children, and tests created for Deaf children are difficult to develop because of the heterogeneity of the population. The challenges to creating rigorous research are numerous in Deaf Education. Nevertheless, there is at least one study out there, that this reviewer knows of, that did use a standardized assessment- for a population in the appropriate age range that was not included in the literature review for this manuscript. Not sure why.

[Reply]: We thank the reviewer for this thoughtful and insightful comment. We fully agree that deaf and hard of hearing (DHH) children represent a highly heterogeneous population, and that linguistic access plays a critical role in shaping cognitive and mathematical development, particularly in early childhood. We also acknowledge the inherent challenges in applying standardized assessments in this population, given that most available tools are normed on hearing children, while instruments specifically designed for DHH learners are difficult to develop due to the variability in language exposure and communication modalities.

In response to this comment, we have revised the Conclusion section to better reflect these methodological and conceptual challenges, emphasizing that mathematical performance in DHH individuals must be interpreted within a broader linguistic and educational framework, rather than relying solely on standardized assessment outcomes.

Regarding the reviewer’s observation about the use of standardized assessments, we agree that this is a critical issue. We have carefully re-examined the literature and identified additional relevant studies that employed standardized or structured assessments in DHH populations, including Kritzer (2009), Pagliaro and Kritzer (2013), and Swanwick et al. (2005). These studies have now been included in the review through a manual screening process and integrated into the analysis. Their inclusion further supports the conclusion that mathematical difficulties in DHH individuals are closely linked to language-mediated processes and task demands, rather than representing a uniform deficit.

We appreciate the reviewer’s suggestion, which has strengthened both the comprehensiveness and the methodological rigor of the manuscript.

____________________________________________________________________

Reviewer #2:

The topic is highly relevant to the fields of mathematics education, special education, and cognitive development. Overall, the study addresses an important gap in synthesizing evidence on domain-specific mathematical abilities and the role of linguistic access in DHH learners.

Major strengths

The review demonstrates several strengths. First, it follows PRISMA 2020 guidelines and uses a predefined protocol registered on OSF, which enhances transparency and reproducibility. Second, the search strategy is comprehensive, covering major databases (PubMed, Scopus, Web of Science) and employing both controlled vocabulary and free-text terms. Third, the inclusion of a structured quality appraisal using JBI tools adds methodological rigor and allows for a nuanced interpretation of study quality. Finally, the narrative synthesis is well-developed and appropriately highlights the heterogeneity of findings, particularly the distinction between linguistic and non-linguistic mathematical tasks.

[Reply]: We thank Reviewer for the positive evaluation of our manuscript and for highlighting the relevance of the topic and the strengths of the methodology and synthesis.

Major concerns

Despite these strengths, several methodological and reporting issues require attention:

Inconsistencies in study selection reporting

There is inconsistency in the number of included studies. The manuscript reports both eight and nine included studies in different sections. Additionally, the PRISMA flow reporting contains numerical inconsistencies (e.g., 579 records minus exclusions does not consistently lead to the reported final sample). These discrepancies should be carefully verified and corrected.

[Reply]: Thank you for your comment. We rechecked the study selection process and corrected the numerical inconsistencies throughout the manuscript. The final number of included studies is 12. We also revised the PRISMA flow diagram and the Results section to ensure consistent reporting of all records identified, screened, excluded, and included.

Clarification of eligibility criteria

The inclusion/exclusion criteria are detailed but somewhat repetitive and occasionally unclear (e.g., overlapping descriptions of study designs). A more concise and structured presentation of PICOS criteria would improve clarity.

[Reply]: Thank you for your comment. The eligibility criteria have been revised to improve clarity and reduce redundancy. In particular, overlapping descriptions of study designs have been removed, and the PICOS framework has been streamlined to provide a more concise and structured presentation.

Lack of quantitative synthesis justification

Although a meta-analysis is not required, the authors should more explicitly justify the absence of quantitative synthesis. Given that some included studies report comparable outcomes, a brief discussion of effect size heterogeneity or feasibility of meta-analysis would strengthen methodological transparency.

[Reply]: Thank you for your comment. A clarification has been added in the Limitations section to explicitly justify the absence of a quantitative synthesis. In particular, we now state that a meta-analysis was not feasible due to substantial heterogeneity across studies in terms of study design, outcome measures, and participant characteristics, as well as the lack of comparable effect size data.

Quality appraisal interpretation

While JBI tools are appropriately applied, the categorization of risk of bias (low/moderate/high) appears partially subjective and not fully standardized. A clearer explanation of thresholds and consistency across different JBI checklists is needed. Additionally, combining qualitative and quantitative studies under similar appraisal logic may require further justification.

[Reply]: Thank you for your comment. In response, the methodological quality assessment process was substantially revised. We removed the use of numerical thresholds and score-based categorizations, as these approaches are not recommended by the Joanna Briggs Institute. Methodological quality is now interpreted using a qualitative framework based on individual JBI appraisal items and key methodological domains rather than predefined cut-off values. In addition, Table 3 was completely redesigned to report the individual JBI items, their ratings (Yes/No/Unclear), and study-specific methodological notes supporting the appraisal process. This approach improves transparency, avoids the use of non-standard scoring systems, and provides a clearer and more detailed justification for the methodological appraisal of each included study.

Heterogeneity of included studies

The included studies vary substantially in design, sample size, age range, and outcome measures. Although this is acknowledged in the discussion, a more structured subgroup interpretation (e.g., by age group, task type, or communication modality) would improve analytical depth.

[Reply]: Thank you for your comment. The Discussion has been revised to provide a more structured interpretation of findings according to key study characteristics, including task type and age group. We also added a statement clarifying that a formal subgroup analysis was not feasible due to heterogeneity, while adopting a structured narrative approach to improve interpretability.

Particularly, we have added the following sentences in the Discussione section:

1) Given the substantial heterogeneity across included studies, findings were interpreted by considering key study characteristics, including age group, type of mathematical task, and communication and educational context.

2) When considering task characteristics, difficulties were more pronounced in mathematically demanding tasks with high linguistic components.

3) When stratified by age, studies in preschool populations consistently highlighted early emerging differences in language-mediated mathematical tasks.

4) Although a formal subgroup analysis was not feasible due to methodological heterogeneity, this structured interpretation provides a consistent framework for understanding variability across studies.

Older studies and methodological limitations

Several included studies are dated (e.g., 1975), and their methodological limitations are acknowledged. However, their influence on overall conclusions should be more critically weighted, as older methodologies may not be fully comparable to contemporary research standards.

[Reply]: Thank you for your insightful comment. We agree that older studies require careful consideration due to differences in reporting standards and methodological practices compared with contemporary research.

In our review, only two studies predate 2000 (1975 and 1995), and both were critically appraised using the JBI tools and interpreted in light of their methodological strengths and limitations. In particular, the study by Ross and Hoemann (1975) presented methodological limitations related to reporting standards, measurement validity reporting, and limited control of potential confounding factors. These limitations were explicitly considered during interpretation of the findings.

Importantly, this issue has been addressed in the revised manuscript. In the Discussion, we now emphasize that older studies were interpreted cautiously and primarily used to provide historical and contextual perspectives rather than to drive the overall conclusions. For example, we added the following statement:

“However, given their methodological limitations, older studies were interpreted cautiously and did not substantially influence the overall conclusions, which are primarily based on more recent evidence.”

We also clarified that the interpretation of the evidence considered the methodological appraisal of individual studies and the consistency of findings across the available literature. Accordingly, conclusions were based primarily on convergent findings across studies presenting generally moderate methodological concerns and relatively consistent results, rather than on isolated findings from older studies.

These revisions ensure a more balanced interpretation of the evidence and acknowledge the methodological limitations associated with earlier research while preserving their contextual value within the overall synthesis.

Language and reporting clarity

Minor grammatical inconsistencies and repetition appear throughout the manuscript (e.g., repeated phrases in Methods and Results sections). A careful language revision is recommended to improve readability and precision.

[Reply]: Thank you for your suggestion. We have carefully revised the manuscript to improve language clarity and readability. Minor grammatical inconsistencies have been corrected, redundant expressions have been reduced, and phrasing has been streamlined, particularly in the Methods and Results sections. In addition, all residual track changes artifacts have been removed to ensure consistency and precision in the final version

Minor comments

The PRISMA flow description should be aligned with the final included study count.

[Reply]: Thank you for your comment. We carefully checked and aligned the PRISMA flow description, figure, and corresponding text

Attachment Submitted filename: REPLY TO REVIEWERS_27.06.2026_FINAL3.docx

# Peer review: Decision Letter 1

**DOI:** 10.1371/journal.pone.0358324.r003

**Authors:** Rana Alkhamra

1 Sep 2026

Mathematical Skills in Deaf and Hard-of-Hearing Children: A Systematic Review

PONE-D-26-21292R1

Dear Dr. Serra,

We’re pleased to inform you that your manuscript has been judged scientifically suitable for publication and will be formally accepted for publication once it meets all outstanding technical requirements.

Within one week, you’ll receive an e-mail detailing the required amendments. When these have been addressed, you’ll receive a formal acceptance letter and your manuscript will be scheduled for publication.

An invoice will be generated when your article is formally accepted. Please note, if your institution has a publishing partnership with PLOS and your article meets the relevant criteria, all or part of your publication costs will be covered. Please make sure your user information is up-to-date by logging into Editorial Manager at Editorial Manager® and clicking the ‘Update My Information' link at the top of the page. For questions related to billing, please contact billing support.

If your institution or institutions have a press office, please notify them about your upcoming paper to help maximize its impact. If they’ll be preparing press materials, please inform our press team as soon as possible -- no later than 48 hours after receiving the formal acceptance. Your manuscript will remain under strict press embargo until 2 pm Eastern Time on the date of publication. For more information, please contact onepress@plos.org.

Kind regards,

Rana Alkhamra

Academic Editor

PLOS One

Additional Editor Comments (optional):

Thank you for submitting the revised version of your manuscript and for providing a comprehensive and thoughtful response to the reviewers’ comments.

Following evaluation of the revised manuscript and your point-by-point responses, I am pleased to confirm that the concerns raised during peer review have been satisfactorily addressed. The revision demonstrates careful consideration of the reviewers’ recommendations and has resulted in substantial improvements in the methodological transparency, internal consistency, clarity, and overall presentation of the work.

Accordingly, I consider the manuscript suitable for publication and am pleased to accept it for publication in the journal.

Reviewers' comments:

Reviewer's Responses to Questions

**Comments to the Author**

Reviewer #3: All comments have been addressed

Reviewer #4: All comments have been addressed

**********

2. Is the manuscript technically sound, and do the data support the conclusions?

Reviewer #3: Yes

Reviewer #4: Yes

**********

3. Has the statistical analysis been performed appropriately and rigorously?

Reviewer #3: Yes

Reviewer #4: Yes

**********

4. Have the authors made all data underlying the findings in their manuscript fully available?

The PLOS Data policy

Reviewer #3: Yes

Reviewer #4: Yes

**********

5. Is the manuscript presented in an intelligible fashion and written in standard English?

Reviewer #3: Yes

Reviewer #4: Yes

**********

Reviewer #3: (No Response)

Reviewer #4: (No Response)

**********

what does this mean?). If published, this will include your full peer review and any attached files.

If you choose “no”, your identity will remain anonymous but your review may still be made public.

**Do you want your identity to be public for this peer review?**  For information about this choice, including consent withdrawal, please see our Privacy Policy

Reviewer #3: No

Reviewer #4: **Yes:**  Alifulloh, Wenda

**********

# Editor report: Acceptance letter

**DOI:** 10.1371/journal.pone.0358324.r004

**Authors:** Rana Alkhamra

PONE-D-26-21292R1

PLOS One

Dear Dr. Serra,

I'm pleased to inform you that your manuscript has been deemed suitable for publication in PLOS One. Congratulations! Your manuscript is now being handed over to our production team.

At this stage, our production department will prepare your paper for publication. This includes ensuring the following:

* All references, tables, and figures are properly cited

* All relevant supporting information is included in the manuscript submission,

* There are no issues that prevent the paper from being properly typeset

You will receive further instructions from the production team, including instructions on how to review your proof when it is ready. Please keep in mind that we are working through a large volume of accepted articles, so please give us a few days to review your paper and let you know the next and final steps.

Lastly, if your institution or institutions have a press office, please let them know about your upcoming paper now to help maximize its impact. If they'll be preparing press materials, please inform our press team within the next 48 hours. Your manuscript will remain under strict press embargo until 2 pm Eastern Time on the date of publication. For more information, please contact onepress@plos.org.

You will receive an invoice from PLOS for your publication fee after your manuscript has reached the completed accept phase. If you receive an email requesting payment before acceptance or for any other service, this may be a phishing scheme. Learn how to identify phishing emails and protect your accounts at https://explore.plos.org/phishing.

If we can help with anything else, please email us at customercare@plos.org.

Thank you for submitting your work to PLOS One and supporting open access.

Kind regards,

PLOS One Editorial Office Staff

on behalf of

Dr. Rana Alkhamra

Academic Editor

PLOS One
